# Millennial-scale climate variability over land overprinted by ocean temperature fluctuations

**DOI:** 10.1038/s41561-022-01056-4

**Published:** 2022-10-31

**Authors:** R. Hébert, U. Herzschuh, T. Laepple

**Affiliations:** 1 Alfred Wegener Institute, Helmholtz Centre for Polar and Marine Research, Potsdam, Germany; 2 Institute of Geosciences, University of Potsdam, Potsdam, Germany; 3 Institute of Biochemistry and Biology, University of Potsdam, Potsdam, Germany; 4 University of Bremen, MARUM – Center for Marine Environmental Sciences and Faculty of Geosciences, Bremen, Germany

## Abstract

Variations in regional temperature have widespread implications for society, but our understanding of the amplitude and origin of long-term natural variability is insufficient for accurate regional projections. This is especially the case for terrestrial temperature variability, which is currently thought to be weak over long timescales. By performing spectral analysis on climate reconstructions, produced using sedimentary pollen records from the Northern Hemisphere over the last 8,000 years, coupled with instrumental data, we provide a comprehensive estimate of regional temperature variability from annual to millennial timescales. We show that short-term random variations are overprinted by strong ocean-driven climate variability on multi-decadal and longer timescales. This may cause substantial and potentially unpredictable regional climatic shifts in the coming century, in contrast to the relatively muted and homogeneous warming projected by climate models. Due to the marine influence, regions characterized by stable oceanic climate at sub-decadal timescales experience stronger long-term variability, and continental regions with higher sub-decadal variability show weaker long-term variability. This fundamental relationship between the timescales provides a unique insight into the emergence of a marine-driven low-frequency regime governing terrestrial climate variability and sets the basis to project the amplitude of temperature fluctuations on multi-decadal timescales and longer.

Improving our ability to characterize internal and natural climate variability is pivotal to improving our long-term climate projections^1^, particularly at the regional scale^2^. The statistics of natural variability can be quantified up to decadal timescales using instrumental observations, but indirect climate proxies are needed for longer timescales.

Proxy-model comparisons aiming to validate climate models generally focus on whether long-term (forced) trends found in proxies can be reproduced by global climate models (GCMs)^3,4^. In this regard, trends over the Holocene from pollen-based climate reconstructions in northern mid-latitudes were shown to be consistent with the forcing and simulated warming in the TraCE-21ka experiment^5^. However, the long-term multi-millennial trend is a rather limited measure that does not evaluate the ability of climate models to simulate realistic climate variability, both forced and internal, at all timescales. Furthermore, climate reconstructions of continental or global average temperatures based on sedimentary proxies generally do not preserve the amplitude of millennial climate variability because of age uncertainty (Methods and Extended Data Fig. 1). To evaluate climate variability across timescales, we consider the power spectral density (PSD, or simply spectrum), *S*(Δ*t*), which provides an estimate of how variance is distributed with frequency, or equivalently with timescale Δ*t*. Others^6^ have provided the classical view of climate variability being dominated by specific quasi-periodic processes, such as solar variations linked to orbital cycles over a mostly random and uncorrelated (that is, white) noise background. Since then, an increasing number of climate proxies and model simulations^7^ have shown a contrasting view of a variance that continuously increases with increasing timescale. Such a behaviour is often well-approximated by a power-law-scaling relationship^8,9^ and can be summarized by the scaling exponent *β* such that *S*(Δ*t*) ~ Δ*t^β^*.

Several studies have suggested that GCMs can simulate realistic climate variability for global and continental mean temperatures across timescales^7,10,11^ that are dominated by the response to external forcing. However, at the regional scale, where only a small fraction of the variability is forced^12^, the capability of GCMs has been called into question for decadal and longer timescales^13–15^. Marine proxies suggest a strong scaling of regional sea-surface temperature (SST) variance from annual to millennial timescales (*β* ≈ 1)^9^ that contrasts with the weaker scaling found in GCMs at longer-than-decadal timescales (*β* < 0.5 in most regions)^15–17^. This leads to an increasing discrepancy between reconstructed SSTs and model simulations at longer timescales, reaching two orders of magnitude in variance at the millennial timescale^8^.

On land, instrumental temperatures show a fundamentally different behaviour compared to the oceans, with only a weak timescale dependency up to multi-decadal timescales (*β* ≈ 0.1)^16–18^. This low-frequency weather has been termed ‘climate noise’^19^ or the ‘macroweather regime‘^20^ and starts at sub-monthly timescales. This difference can be explained by the much smaller heat capacity of land surfaces compared to the oceanic mixed layer^21^. If the macroweather-type behaviour over land held to longer timescales, internal variability would only play a minor role on the uncertainty of regional climate projections at multi-decadal and longer timescales^22,23^. However, a different scaling behaviour of ocean and land at longer timescales would imply an increasingly large variability discrepancy between terrestrial and marine regions, which seems physically implausible given their coupling by the atmosphere, and in contradiction with both diffusive energy balance models (EBMs)^24,25^ and GCMs^17^. This leaves us with the conundrum that we must either reject altogether the marine proxies or see a fundamental change of variability in the terrestrial domain on longer timescales.

Given the limited instrumental record, this fundamental question on the scaling of variability between land and ocean cannot be answered from observations without terrestrial proxy data. Holocene Greenland ice-core δ^18^O timeseries suggest that the weak scaling behaviour found in the instrumental data extends to millennial timescales (akin to white noise with *β* ≈ 0)^26^. However, it is unclear whether the climate variability derived from the high-altitude Greenland ice cores is representative of other terrestrial regions^27^ and to what extent the proxy variability reflects temperature variability^28^.

## Land-temperature variability from pollen records

**Extended Data Fig. 1:**
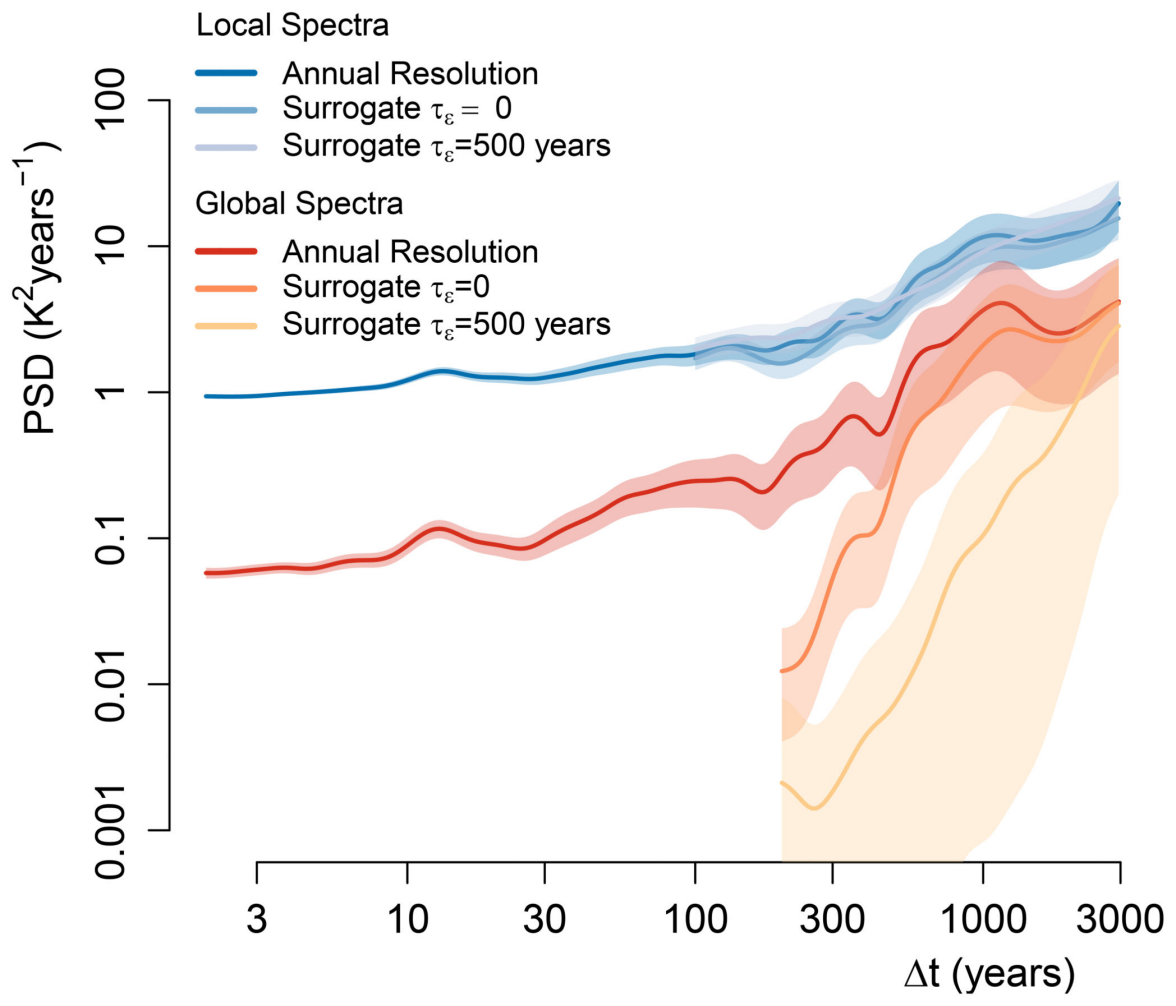
Effect of time uncertainty on the spectral estimates of local and hemispheric temperature. The summer-JJA temperature data of the TraCE-21ka simulation over 0–8 ka BP is extracted at the location of the pollen records to create a surrogate database with and without time-uncertainty (See Methods) from which are computed and shown the mean of all local spectra and the spectra of the hemispheric average temperature; logarithmically spaced axes were used. Only one realization of the surrogate database for each level of time-uncertainty τ_ε_ is shown for simplicity as very similar results are obtained for any given realization. The average spectrum of local temperature variability, which is employed in this study, is robust to irregularity of the pollen records and time-uncertainty. In contrast, the spectra of average (hemispheric) temperature show a strong power-loss due to those effects. This implies that the amplitude of centennial to millennial temperature variations from large spatial scale averages cannot be quantitatively interpreted without the knowledge of the amount and structure of time uncertainty. Shading indicates 90% confidence intervals around the mean.

**Extended Data Fig. 2:**
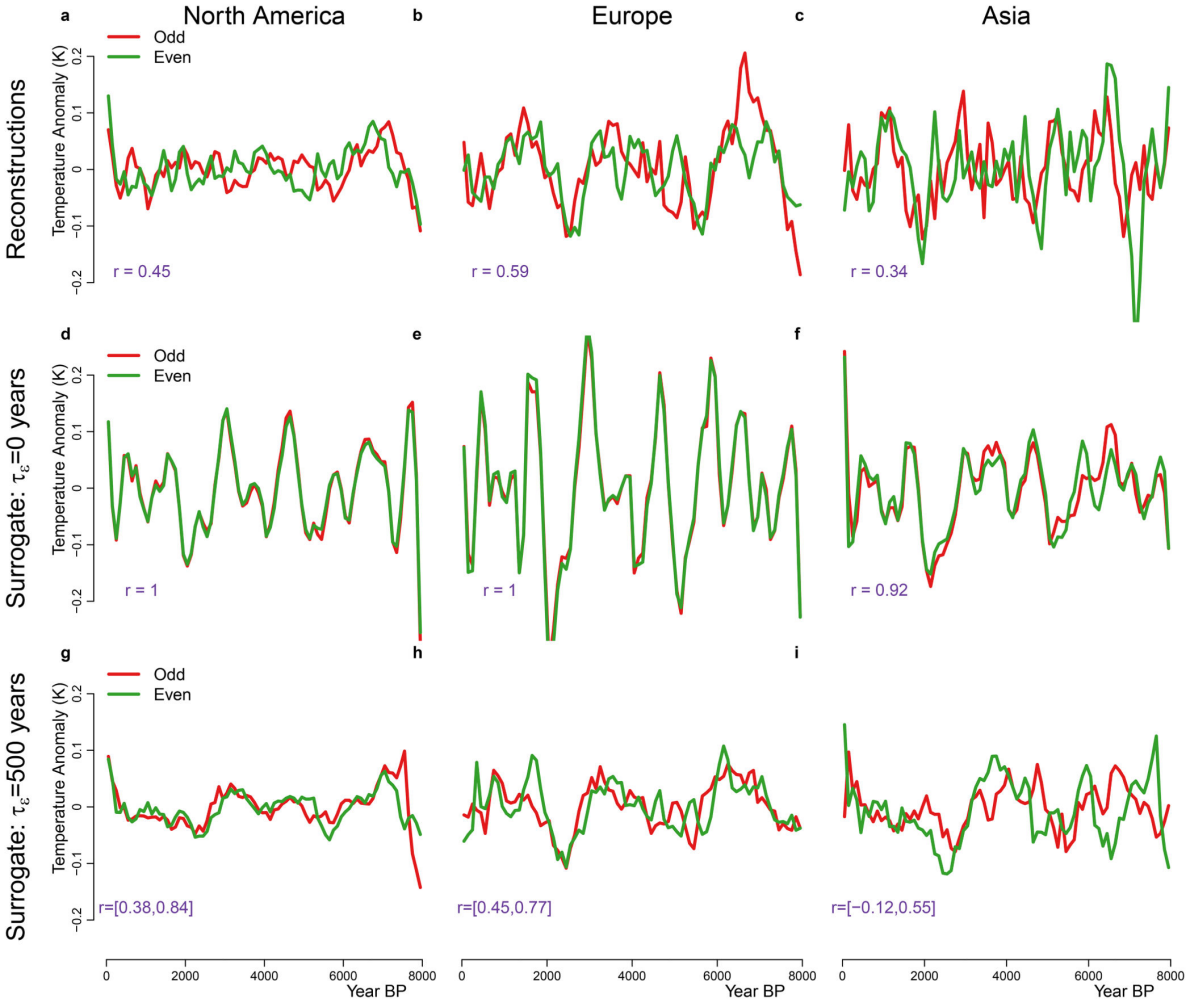
Replicability of summer temperature reconstructions. Timeseries of odd and even sub-groups show good replicability of the millennial scale variability for all continents (the correlation coefficient r is indicated in purple, see Methods). **d,e,f**, To test how the replicability compares to the expectation from time-uncertain irregular timeseries, we repeat the analysis shown in as a,b,c, but using a surrogate database (without time-uncertainty τε) created using the TraCE-21ka simulation (see Methods). **g,h,i**, Same as d,e,f, but for a realization of the surrogate database with time-uncertainty of τ_ε_ = 500 years (see Methods). The 90% range of the correlation obtained for an ensemble of 100 realizations of the 500-year time-uncertainty case is given (purple). The single realization shown was selected because it has correlation close to the mean of the ensemble and is thus representative of the mean behaviour. Comparing d,e,g with g,h,i shows that the replicability of our pollen reconstructions is consistent with the expectation of time-uncertain temperature timeseries. However, the amplitude of the average timeseries should not be interpreted (see Extended Data Fig. 1).

**Extended Data Fig. 3:**
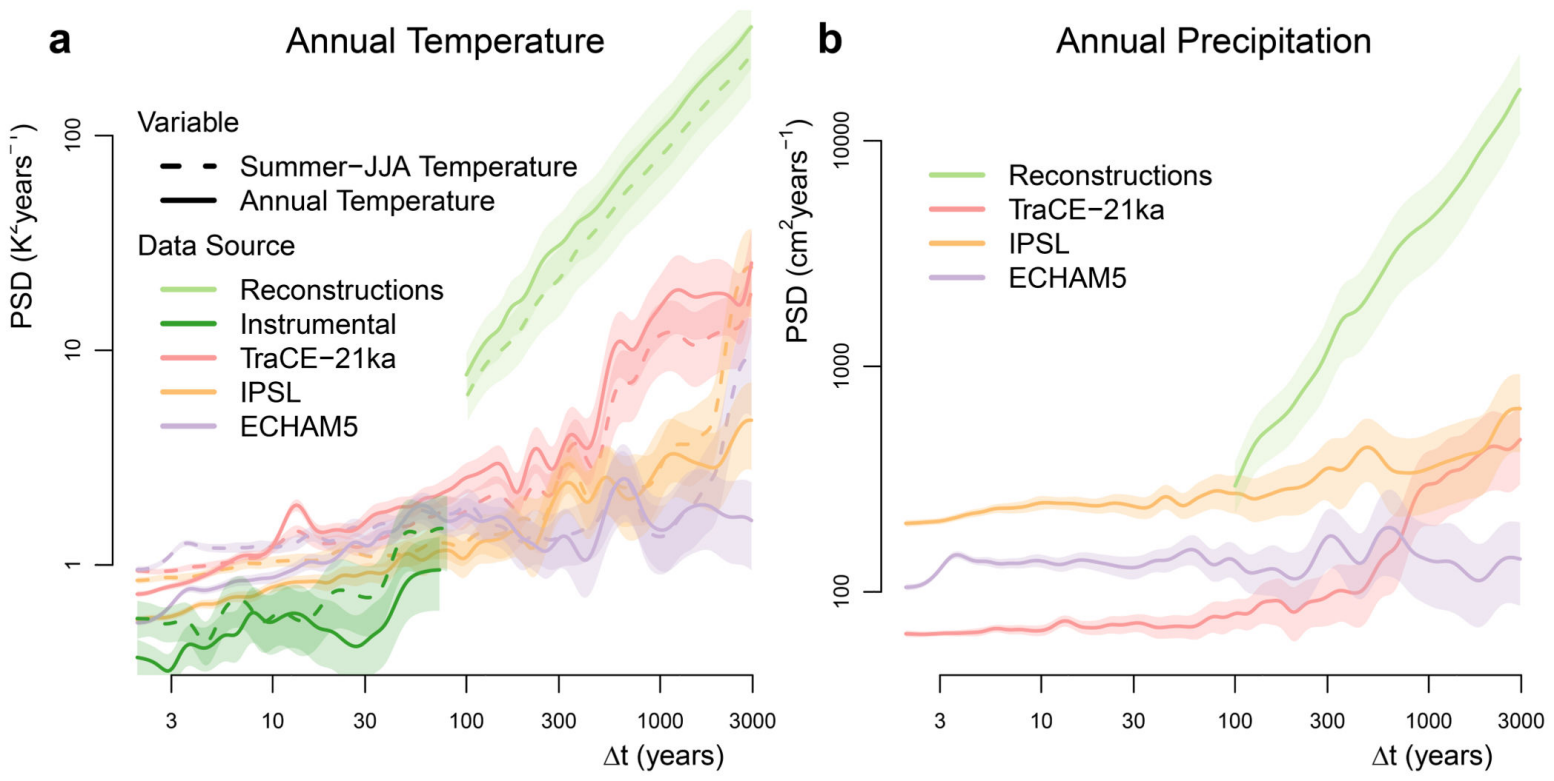
Sensitivity of model-data comparison to the choice of reconstructed variable. a, Comparing the average spectra at the location of pollen records of model simulations, instrumental data (BEST) and reconstructions for the mean summer temperature (dashed) and the mean annual temperatures (solid); logarithmically spaced axes were used. Shading indicates 90% confidence intervals around the mean. The IPSL and ECHAM5 model results exhibit a slightly lower variability in their annual temperature than in their summer temperature over all timescales, except for the longest timescale since it is dominated by leaked power from the Earth’s orbital precession, which mainly affects summer temperature in the Northern Hemisphere during the Holocene. On the other hand, TraCE-21ka generally shows a slightly higher variability in its annual compared to its summer temperature. Although the pollen-based reconstructions calibrated for annual temperature are thought to be less reliable than the summer temperature reconstructions, they give a very similar result. This shows that our conclusions are robust against uncertainties in the seasonal attribution of pollen variability. b, Same as a, but for precipitation instead of summer temperature. While most locations should reflect temperature, here we also tested the boundary case of assuming that all sites reflect precipitation. Even in this extreme case, the main results hold, namely increasing climate variability over land as a function of timescale and a corresponding deficit of variability in the climate models. The three climate models vastly disagree in terms of the amplitude of precipitation variability, but they all show temporal scaling similar to the temperature variability; this is likely caused by the temporal links between precipitation and temperature on long timescales^84^.

To elucidate the behaviour of climate variability over land at timescales longer than centennial, we compiled and analysed an extensive collection of recent Holocene Northern Hemisphere pollen data covering the past 8,000 years, providing the largest spatial coverage of any land-based proxy at millennial timescales. This compilation of pollen data comprises 1,744 unique records from the Northern Hemisphere, 680 of which are from North America, 804 from Europe and 260 from Asia, including the most extensive dataset so far for China^29^ and Siberia^30^. We produced summer (June–July–August (JJA)) temperature reconstructions (hereafter simply termed temperature), as summer temperatures are usually well-correlated with other variables driving vegetation growth^31^, and found a robust signal of millennial-scale variability for all continents (Extended Data Fig. 2). Our results are not sensitive to the choice of summer temperature (Extended Data Fig. 3a) or a potential influence of precipitation (Extended Data Fig. 3b). Pollen-based reconstructions rely on the assumption of dynamical equilibrium between climate and vegetation to calibrate a transfer function based on the modern distribution of vegetation and the associated spatial gradient in the climatology. This assumption is timescale-dependent and is generally more valid at longer timescales^31^. In this respect, millennial-scale estimates of temperature variability, on which our quantitative results hinge, should thus be particularly robust, especially considering there is evidence that vegetation responds on timescales of centuries or faster^32,33^. Finally, we verified that the estimates of millennial variability were not systematically biased by human influences (Supplementary Fig. 2) and that our estimates are robust to the time uncertainty and irregular spacing of the timeseries (Extended Data Fig. 1).

**Extended Data Fig. 4:**
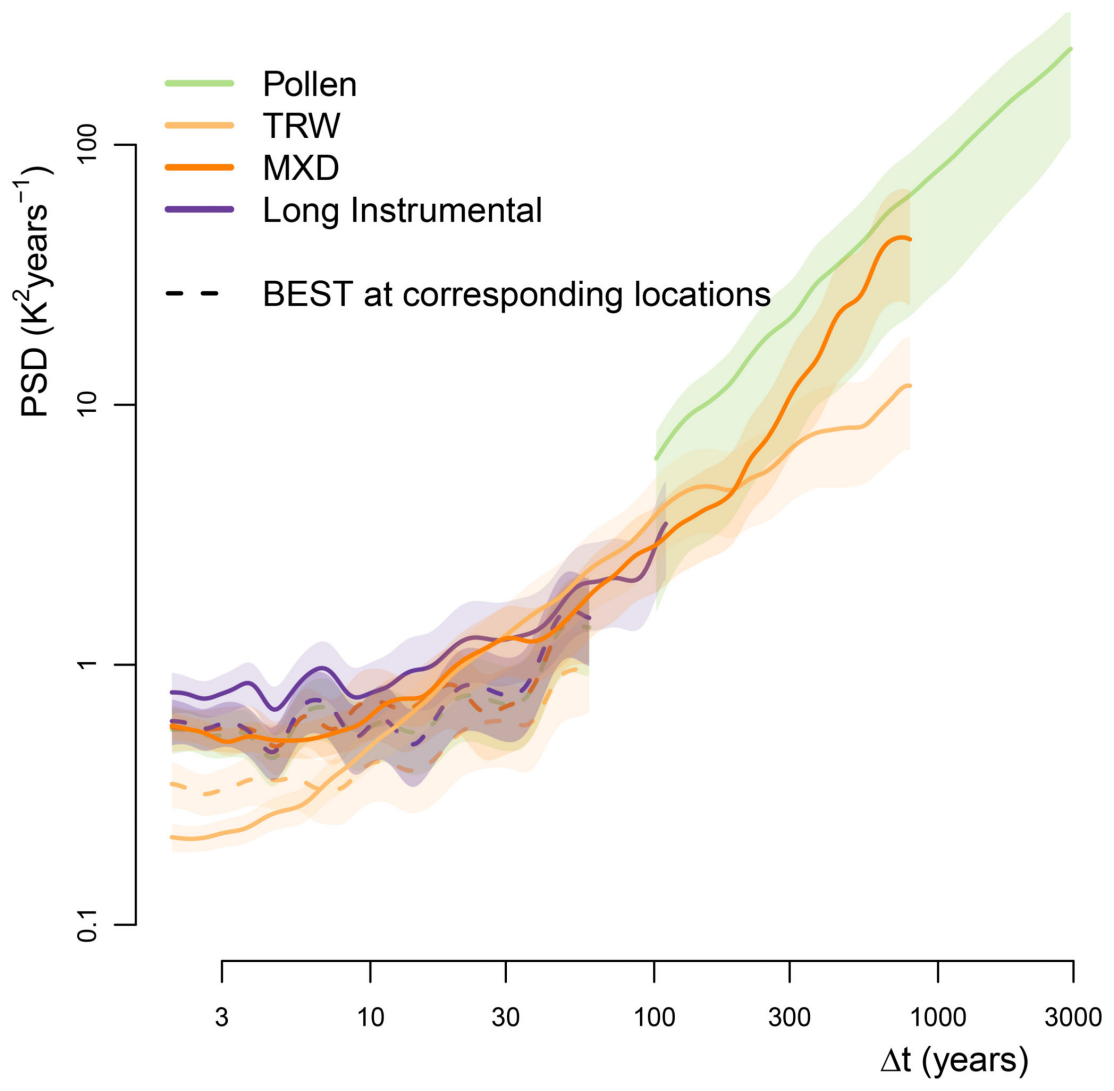
Spectral estimates from tree ring based and long instrumental temperature series. To test whether the transition from the weakly scaling macroweather regime to the stronger scaling oceanic regime is also visible from independent temperature reconstructions, we show the spectral estimates of land temperature variability from tree ring width (TRW), maximum latewood density (MXD) measurements and long (>170 years) instrumental series together with the pollen-based reconstructions (solid); logarithmically spaced axes were used. The spectral estimates from the BEST gridded instrumental data is provided as reference at the corresponding locations (dashed, detrended with respect to log(CO_2_)). The MXD and long instrumental spectra support the scaling of the pollen data. The TRW spectra deviates at high and low frequencies due to the known biases of this proxy^85–87^ (see Supplementary Information Note 1).

**Fig. 1:**
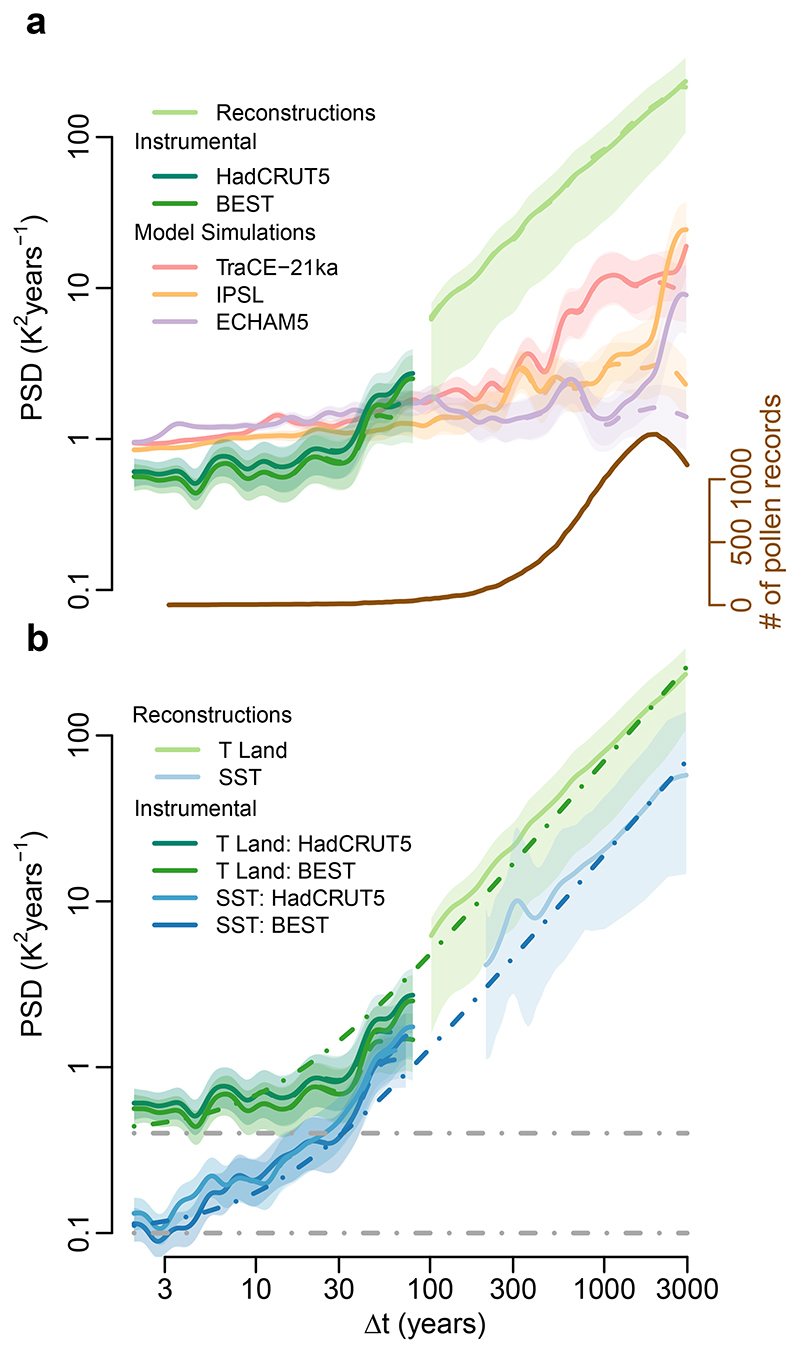
Average spectral estimates of local land temperature over the Northern Hemisphere. **a**, PSD estimates of land air temperature (*T* land) from pollen-based reconstructions along instrumental data and model simulations, both extracted at the pollen record locations; logarithmically spaced axes were used. Also shown are estimates from timeseries detrended with a 23-kyr sinusoidal or with respect to log(CO_2_) (dashed). The number of pollen records contributing to each timescale is indicated below (brown axis). **b**, Average spectral estimates from reconstructed annual SSTs^9^, and instrumental data at the corresponding locations. Observational spectra from **a** are reproduced. Linear combinations of power laws with slope *β* = 1.2 and white-noise series are shown as dashed-dotted lines (land in green, sea in blue and white-noise levels in grey). Shading indicates 90% confidence intervals around the mean.

Pollen-based reconstructions, once coupled with instrumental data^34^, enable us to establish a comprehensive picture of regional land-temperature variability from inter-annual to millennial timescales (Fig. 1 and Methods). The spectrum of instrumental air temperature shows a rather flat scaling behaviour that is characteristic of the macroweather regime. At longer timescales, however, the pollen-based reconstructions show a strong increase in variability with increasing timescale (Fig. 1a). This suggests that, even in the relatively stable recent Holocene, there exists substantial centennial- to millennial-scale temperature variability. Although human influences have historically impacted vegetation composition, they do not dominate this result, as they were not found to systematically bias millennial-scale temperature variability (Supplementary Fig. 2). This behaviour clearly differs from the rather flat macroweather regime and resembles power-law scaling with an exponent *β* near one for timescales longer than centennial. Traces of this increased scaling behaviour at multi-decadal timescales already appear in the instrumental record, and it is corroborated by dendrochronological timeseries and long instrumental series (Extended Data Fig. 4 and Supplementary Note 1).

**Extended Data Fig. 5:**
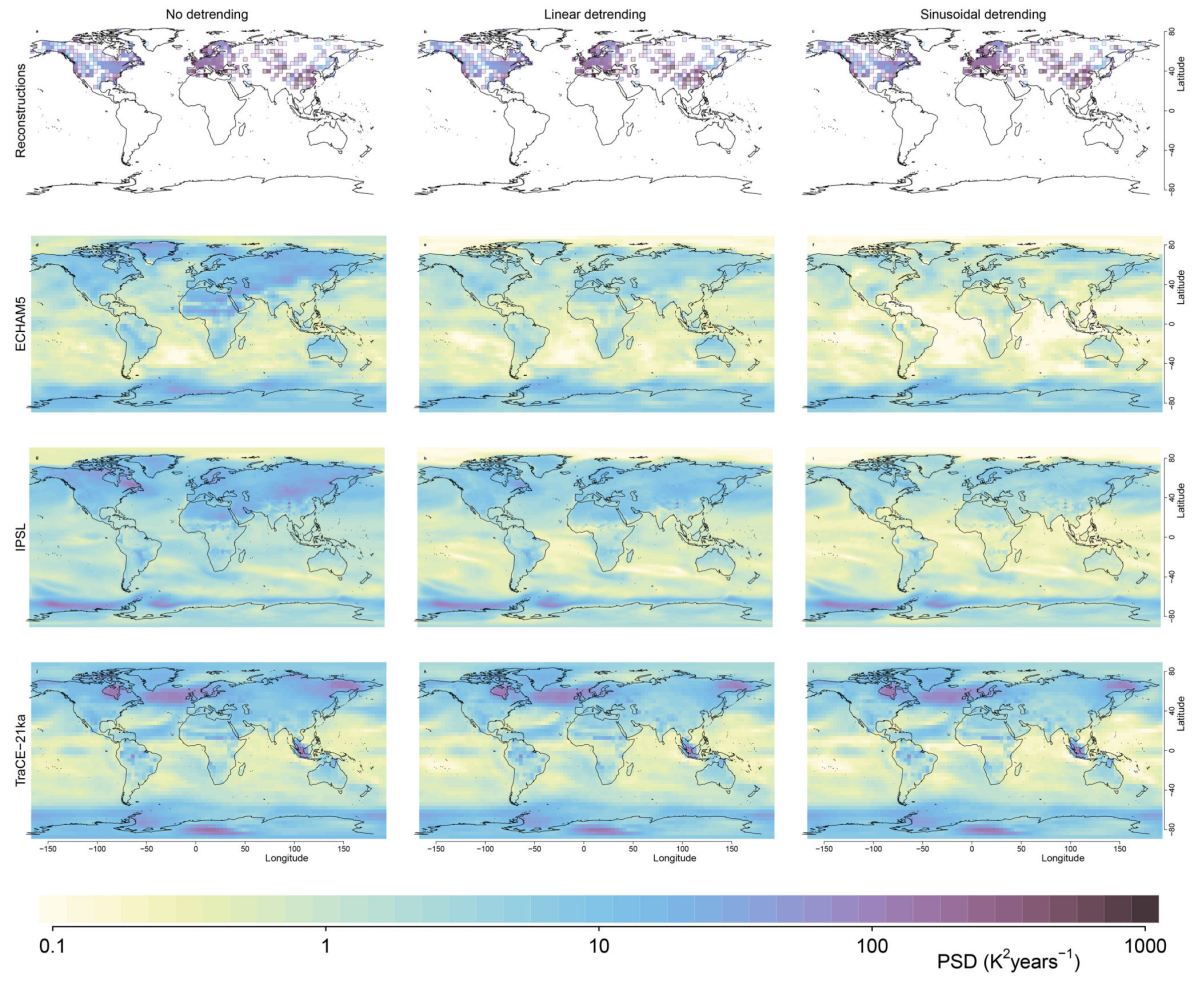
Comparison of millennial scale variability in the reconstructions and models. **a-c** Millennial temperature variability PSD_1000-3000 years_ (mean PSD for the timescale band 1000-3000 years) for the pollen-based reconstructions in 2°×2° spatial bins. **d-l** Millennial temperature variability PSD1000-3000 years for the three climate models (without smoothing). The results with different detrending methods before computing the power spectra are compared: **d,g,j** without detrending, **e,h,k** with linear detrending and **f,i,l** with a 23-kyr sinusoidal detrending. The same colour scale, using logarithmic spacing, is used for all maps. The model simulations generally show 10-100 times less variability than the reconstructions.

**Extended Data Fig. 6:**
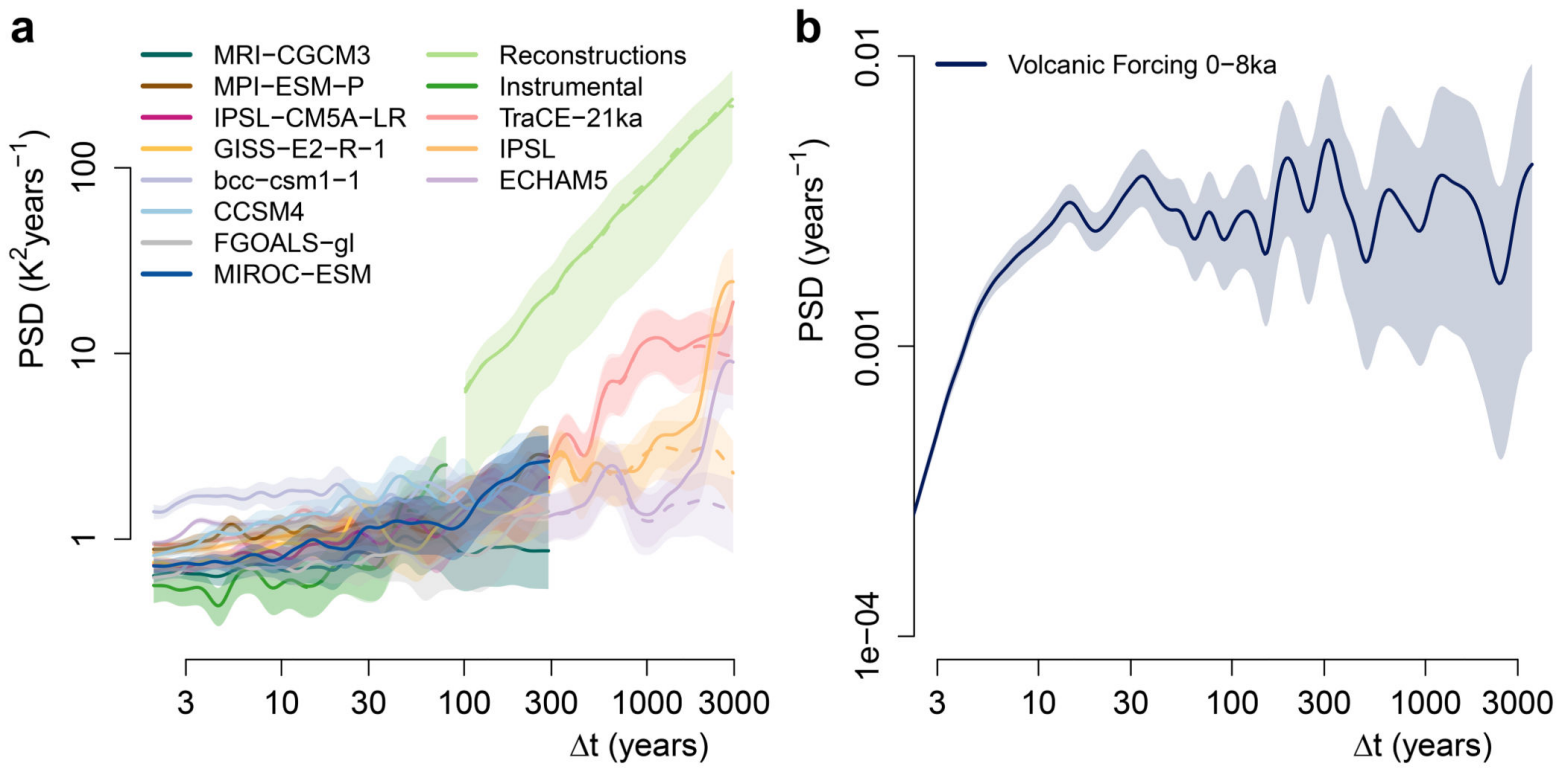
Impact of forcing on the model-proxy comparison. **a**, Same as Fig. 1a, but with the local spectra of summer temperature variability of 8 fully-forced (that is including volcanic and solar forcing) millennium simulations added. Only the BEST instrumental spectra are reproduced for simplicity. **b**, Spectrum of the reconstructed aerosol optical depth, that is the volcanic forcing, over the late Holocene (0–8ka)^88^. The fully forced simulations show similar local variability as the long Holocene simulations. As the volcanic forcing spectrum is flat on multi-decadal to millennial time-scales, missing volcanic forcing in the Holocene is unlikely to fully reconcile the model-proxy variability mismatch unless the amplitude of the response and its response time are both severely underestimated in the climate models. There are indications however that volcanic forcing could partially reduce the variability mismatch^89^. Shading indicates 90% confidence intervals around the mean.

We benchmark our result against three transient climate-model simulations of the recent Holocene: IPSL^35^, ECHAM5^36^ and the last 8,000 years of the TraCE-21ka deglaciation experiment^37^ (that is, after the last freshwater forcing events). The transient simulations of these GCMs show the weak scaling behaviour characteristic of the macroweather regime, although with higher annual to decadal variability relative to the instrumental data, as previously recognized^17^. However, they fail to capture the increase in variability observed in the reconstructions at multi-decadal timescales. Instead, they show a relatively weak increase at multi-centennial timescales and a sharp increase at millennial timescales due to spectral leaking from the orbital 23-kyr precession cycle (Fig. 1a and Extended Data Fig. 5). As a result of this divergence in variability scaling, there is an increasing deficit in temperature variability observed in the simulations compared to the reconstructions. The range of variance ratios between the reconstructions and the different model simulations increases from 6–8 over the 100–300 years timescale to 11–54 (12–85 with orbital detrending) over the 1,000–3,000 years timescales band, resembling the discrepancy between models and proxy data for regional SST variability^9^. Similar results are also obtained with the fully forced Coupled Model Intercomparison Project – Phase 5 (CMIP5) simulations of the last millennium, and accounting for volcanic forcing over the Holocene is unlikely to resolve this discrepancy (Extended Data Fig. 6).

## Marine influence on the spatial pattern of millennial variability

Comparison of the reconstructions over land with estimates of marine variability^9^ shows a very similar low-frequency scaling behaviour (Fig. 1b). The observed parallel scaling behaviour is expected if both components vary more coherently as climatic variability becomes a global phenomenon over longer timescales^24^, as also indicated by coherent land and ocean average temperatures^5^. EBMs suggest that this parallel behaviour of land and oceans on long timescales is due to heat exchange between the land and ocean compartments. In such models, land air temperature can be described as a linear combination of the SST and a time-dependent forcing over land^25,38^; the resulting variability spectrum over land is then a linear combination of the spectra of each term (Fig. 1b) when the two are uncorrelated (Supplementary Note 2). In this framework, the change in scaling behaviour can be regarded as a transition from the macroweather regime at shorter timescales, dominated by a weakly scaling forcing component akin to white noise over land^21^, to an oceanic regime dominated by the SST component at timescales longer than decadal. Interestingly, the parallel behaviour between land- and ocean-temperature spectra on multi-decadal to millennial timescales provides no evidence for additional terrestrial slow climate feedbacks. The oceanic component present in land-temperature variability appears amplified by a factor of ~4 in PSD or ~2 in amplitude (Fig.1b). This factor is similar to the land–sea warming contrast^39^ observed during the last century^40^ and within the range of land–sea warming ratios measured in GCMs^41^. This is thought to be the result of local feedbacks, such as evaporation feedback, when moisture availability over land limits evaporative cooling in comparison with marine regions^42^, and also because of the asymmetry in the land–ocean heat exchange, which favours land due to its lower specific humidity^41^.

**Fig. 2:**
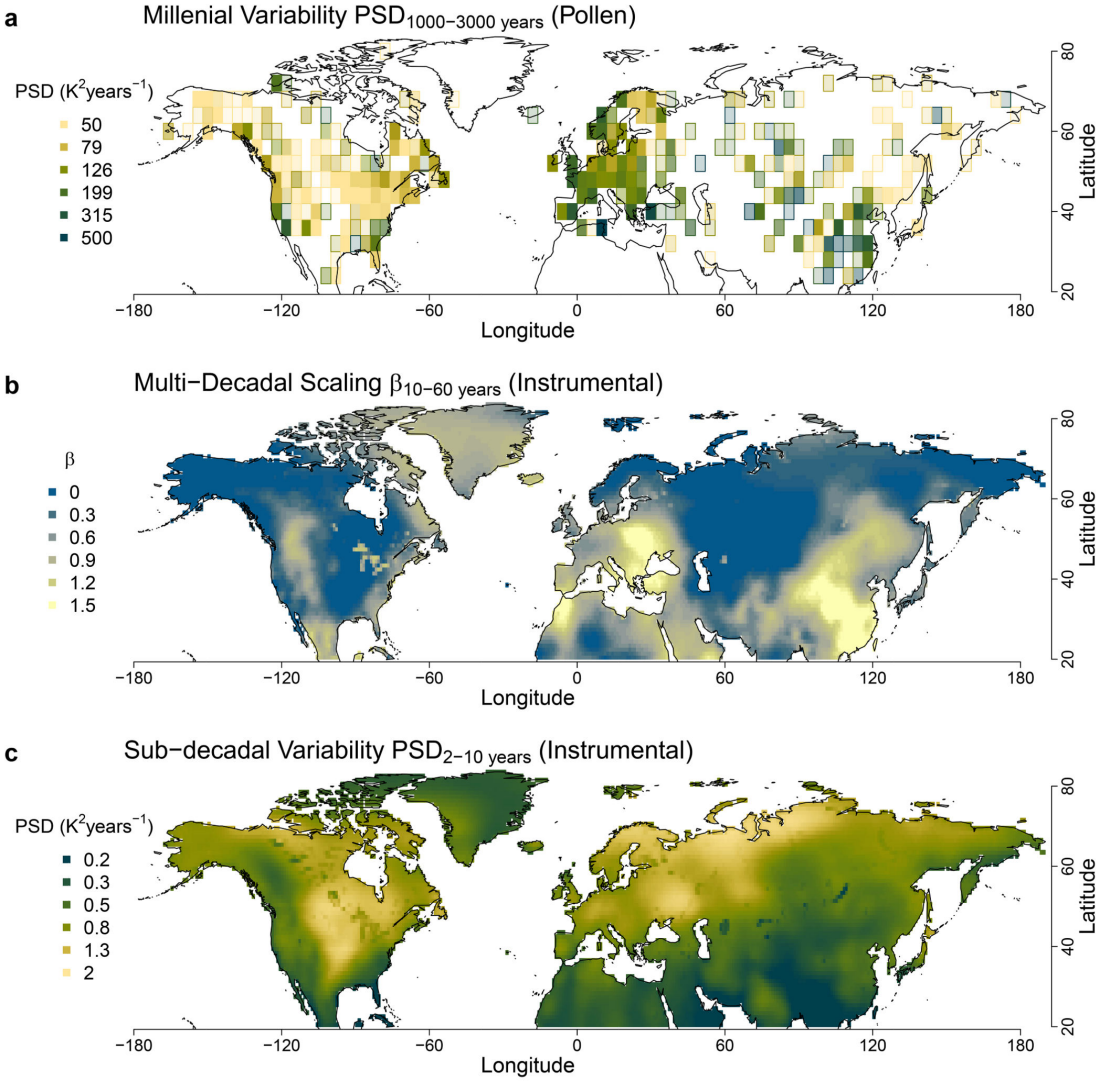
Spatial patterns of temperature variability in instrumental data and pollen-based reconstructions. **a**, Map of millennial variability estimated from the summer temperature spectra of pollen-based reconstructions as the mean PSD over the 1,000–3,000-yr timescale band in 4° × 4° spatial bins with opacity linearly proportional to the number of records in the bin and saturating when there are five records (see Extended Data Fig. 7 for raw and smoothed estimates).**b**, Map of the multi-decadal scaling exponent *β* from the spectra of instrumental temperature records fitted over the 10–60-yr timescale band. **c**, Map of sub-decadal variability, mean PSD over the 2–10-yr timescale band, estimated from the spectra of instrumental temperature records. A logarithmically spaced colour scale was used for **a** and **c**.

The extensive spatial coverage of pollen-based reconstructions allows us to perform a spatial analysis of the millennial-scale temperature variability PSD_1,000–3,000 years_ (the mean PSD over 1,000–3,000 years; Fig. 2a) and investigate the potential link to oceanic influence. The spatial coherence (Moran’s *I* = 0.2, *P* < 0.001; Methods) shows that the variability estimates are not drowned out by local noise. Over Europe, millennial-scale variability decreases inland along the path of prevailing winds blowing from the Atlantic Ocean, and is lowest over Fennoscandia (Finland and Scandinavia), where blocking events are most frequent^43^. Similarly, China’s high millennial variability is linked to variability in the East Asian Summer Monsoon^44^, and further north in eastern Siberia the dominant westerlies bring little oceanic influence. This further suggests that higher millennial variability relies on higher connectivity to oceans, as implied by EBMs, although compounded by local sensitivity. The high variability in central Asia remains an outlier given the strong continentality there, but the significance is lower because of the sparseness of records. It is also possible that the lower connectivity to oceans is compensated by the stronger local climate sensitivity^40^, which may be linked to hydrological feedback due to the arid conditions^42^ and to snow-albedo feedback at higher elevations^45^. Meanwhile, in North America, the lowest millennial variability is found in the prairies, near the centre of the continent, where the westerlies predominantly blow from the northwest, and the oceanic influence is lowest.

## Links in the continuum of land-temperature variability

**Extended Data Fig. 7:**
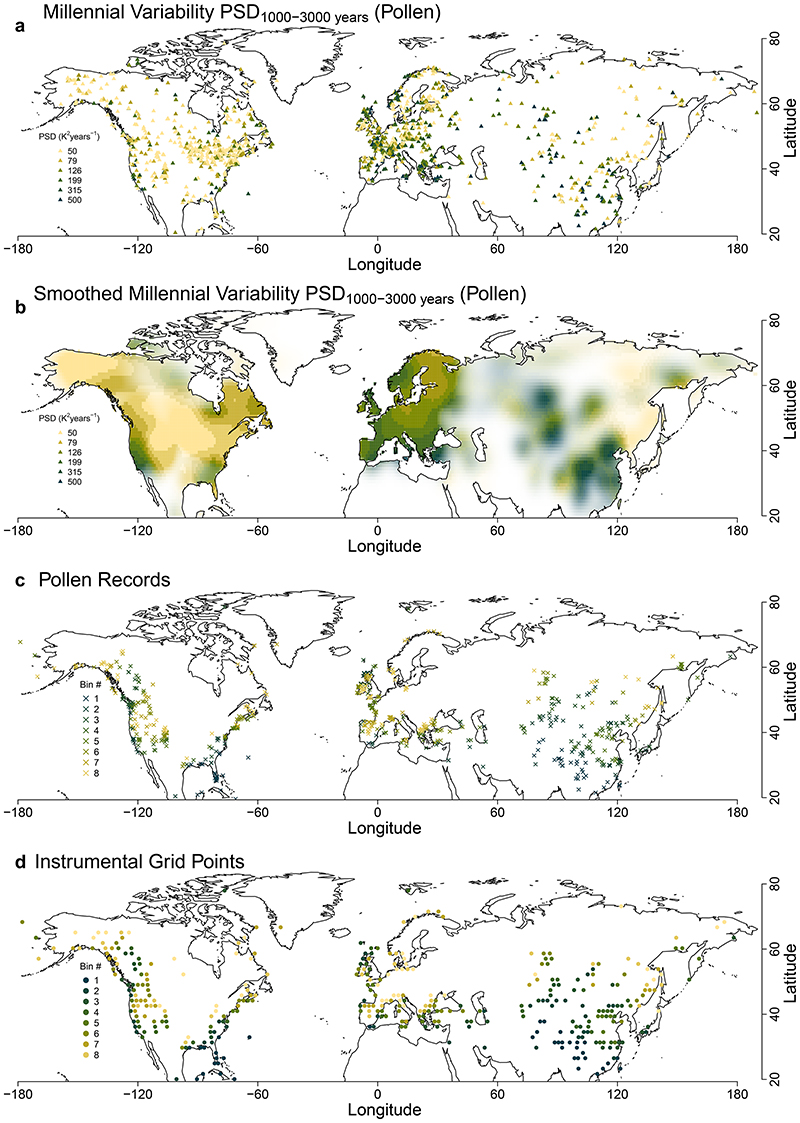
Raw and smoothed maps of pollen-based estimates of millennial temperature variability, and location of the records contributing to each spectral bin. **a**. Same as Fig. 2a, but for the individual sites. **b**. Same as a but after applying a 300-km Gaussian smoothing. The opacity is proportional to the effective number of records contributing to the smoothed value, that is the sum of the Gaussian weights, and is fully opaque when 5 or more effective records were available. **c**. Shown are the location of individual pollen records that were considered in the analysis. The colours correspond to those in Fig.3, indicating which records are included in the binning of each spectrum. **d**. Shown are the grid points of the instrumental dataset that are near pollen records. The colours also indicate the corresponding bins as in **a** and Fig. 3. Each of the 8 non-overlapping bins contain 78 grid points based on the sub-decadal variability (see Methods Sub-Decadal Variability Binning).

**Extended Data Fig. 8:**
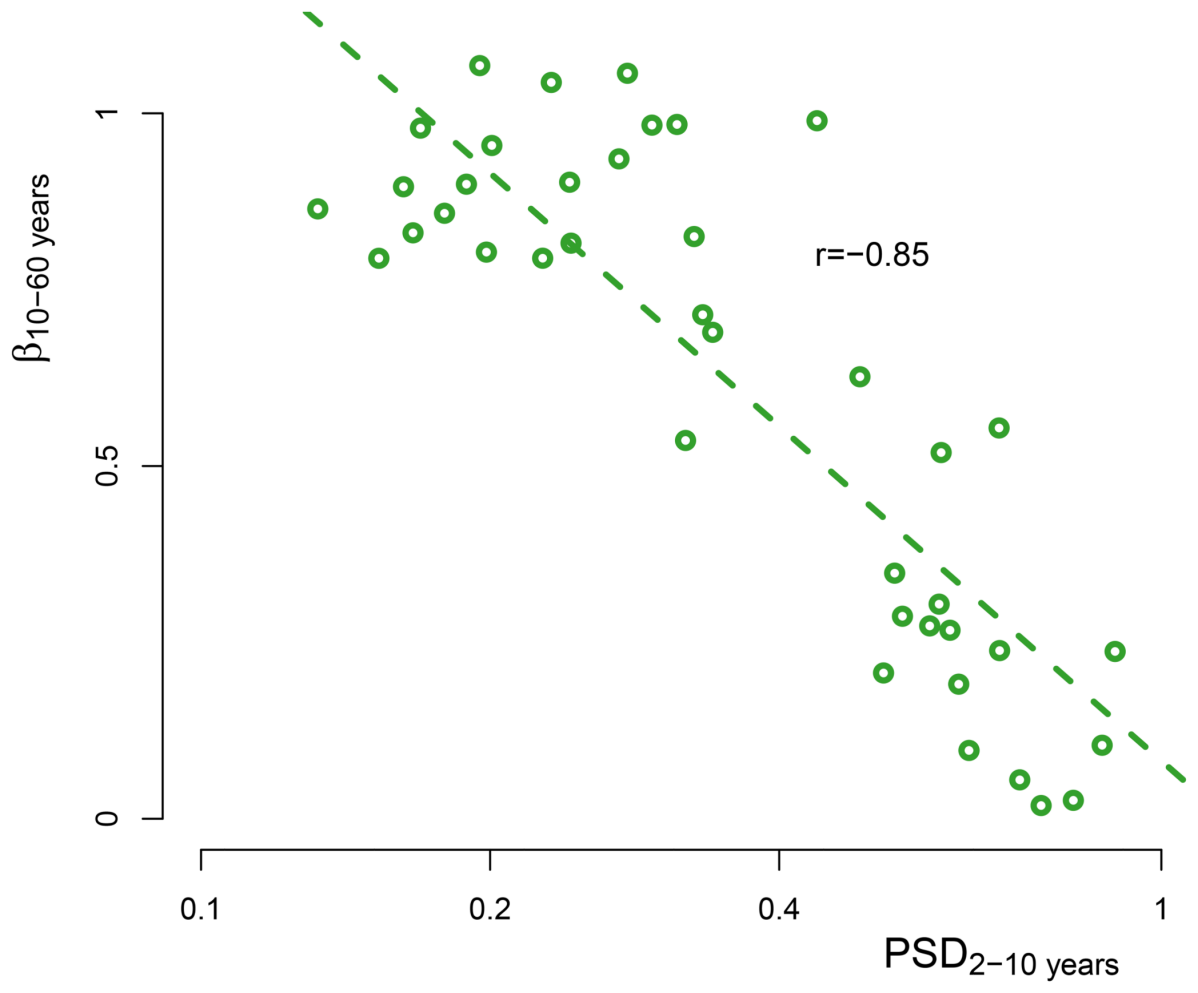
Relationship of the sub-decadal variability and multi-decadal scaling for land extratropics. The correlation plot between the sub-decadal variability (2–10 years) and the multi-decadal scaling (10–60 years) over land for all grid points north of 20 N; the x-axis is logarithmically spaced. We use 40 bins to have a similar number of grid points per bin as Fig. 3; the results are insensitive to this choice.

**Fig. 3:**
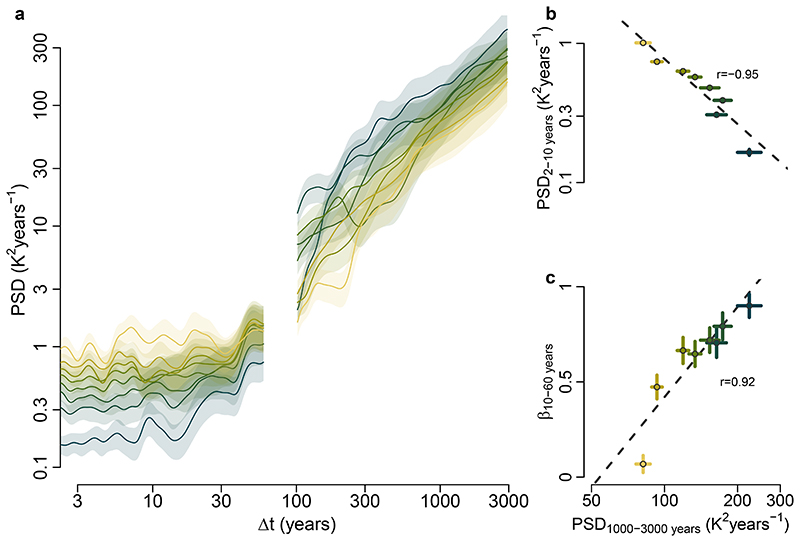
Spectral estimates of land temperature as a function of sub-decadal variability. **a**, PSD estimates of the instrumental temperature (Δ*t* < 60 years) and of the pollen-based reconstructions (Δ*t* > 100 years) binned according to sub-decadal instrumental variability (Methods and Extended Data Fig. 7c,d); logarithmically spaced axes were used. Shading indicates 90% confidenceintervals around the mean. **b**, Relationship of the instrumental sub-decadal temperature variability PSD_2-10years_ and the pollen-based millennial temperature variability PSD_1,000-3,000 years_. Vertical and horizontal lines indicate the standard error of the mean within each bin. **c**, As in **b**, but between *β*_10-60years_ and PSD_1,000-3,000_ years.

We use the instrumental data to study the mechanisms governing the spatial distribution of the millennial variability and the continuum of variability. The scaling of variability in instrumental data has already been shown to be related to the strength of the annual cycle and of the sub-decadal variability^8^. If we aggregate the instrumental data and reconstructions based on the sub-decadal variability PSD_2–10 years_ (the mean PSD over 2–10 years; Fig. 3 and Extended Data Figs. 7 and 8), a clear relationship appears with the emergence of the low-frequency regime, quantified by the multi-decadal scaling exponent *β*_10-60 years_ (*β* regressed over 10–60 years): locations with lower sub-decadal variability thus show a stronger increase of variability towards longer timescales, as indicated by higher multi-decadal scaling (Figs. 2b,c and 3 and Extended Data Fig. 8). We should thus expect an inversion where regions of low sub-decadal variability, typically characterized by more maritime influences, would become regions of high variability at longer-than-centennial timescales. Similarly, continental regions characterized by high sub-decadal variability would be expected to become regions of relatively low variability on longer timescales.

**Extended Data Fig. 9:**
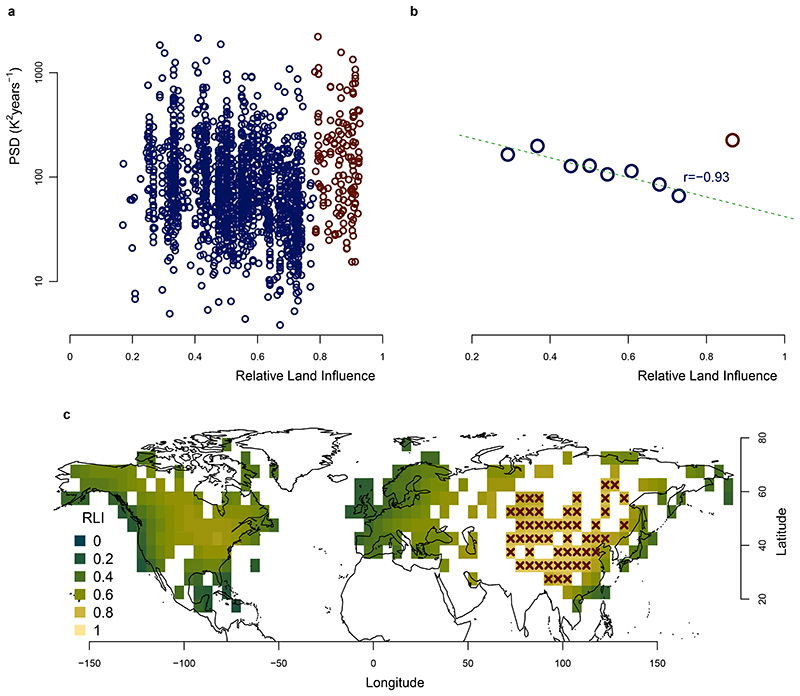
Millennial variability and relative land influence. **a**, The millennial variability PSD_1000–3000 years_ is shown as a function of the relative land influence (RLI) index^46^. The points with RLI values above 0.78 were indicated in red to underline the change in behaviour. **b**, The pollen records were binned according to the RLI into 8 groups of 198 records, and PSD_1000–3000 years_ was calculated from the average of their spectra (blue). A ninth distinct group comprising the 158 records belonging to areas where the RLI was above 0.78 was considered (red). The correlation given (blue) does not include the ninth group since it is an outlier as discussed in the manuscript and methods. **c**, Map of the RLI for grid boxes containing pollen records. The grid boxes with RLI > 0.78 are marked with red crosses in order to highlight the regions in the ninth bin.

Indeed, this hypothesized relationship is confirmed by the pollen-based reconstructions. Their estimates of millennial temperature variability PSD_1,000–3,000 years_ show a strong anti-correlation with the sub-decadal variability PSD_2–10 years_ (*r* = −0.95, *P* < 0.03; Fig. 3b) and a strong correlation with the multi-decadal scaling *β*_10–60 years_ (*r* = 0.92, *P* < 0.01; Fig. 3c). These substantial strong relationships between the pollen-based reconstructions and independent instrumental temperature data demonstrate a fundamental link of temperature variability from sub-decadal to millennial timescales. The spatial pattern of the variability (Fig.2 and Extended Data Fig. 5) further suggests that this relationship is caused by a varying marine influence. This is supported by a strong correlation (*r* = -0.93, *P* < 0.02) of the spatial pattern of millennial variability with the relative land influence index^46^ (RLI; ‘Relative land influence index’), which quantifies the relative contributions of land and ocean to atmospheric fluxes at a given location using a Lagrangian trajectory model (Extended Data Fig. 9). An exception is Central and East Asia, where the RLI is highest, but millennial variability remains high. Finally, our result provides continentality as a complementary explanation for the relationship between the amplitude of the annual cycle (an indicator for continentality) and the inter-annual variability scaling in the instrumental relationship proposed in ref. ^8^. Therefore, our findings complete the linkage between seasonal and millennial land-temperature variability.

## Discussion

Our results indicate that current GCMs underestimate regional temperature variability over land at timescales longer than multi-decadal (Fig. 1a and Extended Data Fig. 5).In combination with the spatial pattern of variability (Fig. 2), this suggests that the deficit in low-frequency variability is related to an underestimation of marine variability^9^. The interpretation of climate-sensitive proxies remains an area of active research, and, in principle, it remains possible that the observed model-data mismatches stem from non-climatic variability. However, several lines of evidence argue against this interpretation. First, as yet, there are no known archival processes that could artificially create such power-law scaling in sedimentary archives^47^. Specifically, known processes such as counting errors, spatial or temporal aliasing, and bioturbation in the sediment cannot explain the power spectra of variability found here (Figs. 1 and 3). The vegetation’s response time to climate can affect the variability, but it would only reduce it on the faster timescales, in contrast to increasing it on millennial timescales. Second, the consistency between independent marine and terrestrial archives (Fig. 1b) provides further support for the temperature-variability reconstruction. Although, in principle, similar non-climatic effects could create scaling in both sedimentary archives, in each case, annually resolved independent archives (corals for SST^9^, tree records for land temperature; Extended Data Fig. 4) confirm the scaling behaviour and amplitude. Finally, the spatial relationship with the independent instrumental temperature data (Fig. 3) also indicates that this is no artefact from the proxy data.

Thus, pollen-based reconstructions support the paradigm of an increasing continuum of climate variability with increasing timescales^8^, in contradiction with the local temperature variability in current GCM simulations and the classical picture of ref. ^6^. More importantly, our results extend previous findings from instrumental data^8^ and demonstrate a fundamental link between inter-annual, multi-decadal and millennial timescales driven by the interaction of marine and terrestrial temperature variability modulated by continentality.

This fundamental behaviour of temperature variability has implications for the relative impact of natural and anthropogenically forced variability. High-latitude regions characterized by high inter-annual variability show a weaker oceanic regime and, ultimately, less natural variability on millennial timescales. As these regions are also highly sensitive to anthropogenic forcing, the impact of anthropogenic warming, relative to natural variability, will be greater. However, regions of strong maritime influence, where most of the world’s population is located, could see large natural variability that is not simulated by current GCM projections, which tend to display monotonous warming^40^. It is thus possible that, until now, the stronger natural variability at multi-decadal timescales in maritime regions has partly overshadowed the anthropogenic warming in those regions, which could explain their lower observational transient climate sensitivity^40^. Integrative archives such as glaciers should be particularly sensitive to this increased memory^18^ and could be used to verify our findings. Large compilations of climate archives have the potential to inform us on the spatial patterns of slow variability and their underlying causes. Further studies combining multiple proxies over land and ocean, and the development and inclusion of proxy system models^48^ for a more direct model-proxy comparison, show great promise to improve our understanding of the spatio-temporal correlation structure of climate variability.

## Online content

Any methods, additional references, Nature Research reporting summaries, source data, extended data, supplementary information, acknowledgements, peer review information; details of author contributions and competing interests; and statements of data and code availability are available at https://doi.org/10.1038/s41561-022-01056-4.

## Methods

### Reconstructions

The fossil dataset LegacyPollen 1.0^49^ includes taxonomically harmonized fossil pollen data from North America and Europe obtained via the Neotoma Palaeoecological Database^50^, and from Asia combining refs. ^29^ and ^30^. The datasets were combined, keeping the 70 most common taxa according to Hill’s second number. A fossil database comprising 1,744 records was retrieved based on the requirement that the resulting spectral estimates covered timescales of at least one-fifth of an order of magnitude, that is, 0.2 in a base-10 logarithm, below one-half the length (to avoid the well-known multitaper low bias at long timescales)^51^. Extreme outliers (with logarithm of PSD_1,000–3,000 years_ more than four standard deviations away from the mean of all records) were discarded (*n* = 5), but their inclusion did not affect any of our conclusions.

The method and data for calibration agree with those of ref. ^52^. The weighted averaging partial least square (WAPLS)^53^ method was used to calibrate transfer functions relating the pollen assemblages to the summer temperature, with leave-one-out cross-validation to assess the model performance. The WAPLS method exploits the modern spatial relationship between a modern calibration dataset of pollen assemblages and the climatology to calibrate a transfer function that is then applied on the fossil assemblages to reconstruct timeseries. The modern climate data used for calibration were the average of the JJA climatologies, that is, the summer temperature, for the years from 1970 to 2000 as obtained from WorldClim 2.1^54^. The modern pollen dataset used for calibration consists of 15,379 sampling sites. For each fossil record location, we selected a unique subset of modern sites within a 2,000-km radius so as to reduce the impact of false analogues^55^; if there were fewer than 30 samples within the radius, the reconstructions were not performed and the record discarded. The pollen percentages of both the modern and fossil databases were square-root-transformed to decrease the dominance of abundant taxa with high productivity. The number of retained WAPLS components was selected using a randomization *t*-test based on the criterion that adding a component should improve the root-mean-square error by at least 5%^56^. The transfer function with the retained number of components was then used to predict the summer temperature of the fossil assemblages, that is, the reconstructions. For the sensitivity tests, the same method was also applied to reconstruct annual mean temperature and annual precipitation (Extended Data Fig. 3), also using the climatologies from WorldClim 2.1^54^ for the calibration of each variable.

### Robustness of the reconstruction and RandomTF test

To test the robustness of the summer-temperature reconstructions, we performed replicability experiments following the methodology in ref.^5^. For each continent, the reconstructions were divided into two sub-groups (odd and even indices after ordering them according to longitude) and averaged in the temporal domain after linear interpolation at a resolution of 100 yr and detrended using a 6.5-kyr LOESS filter. The result shows replicability of the millennial-scale variability for all continents, with good correlation between the odd and even sub-groups: *r* = 0.45 for North America, *r* = 0.59 for Europe and *r* = 0.34 for Asia (Extended Data Fig. 2a–c). These values are within the expectation for surrogate data with time uncertainty (Extended Data Fig. 2d–i and ‘Surrogate data and time uncertainty’).

Following ref.^57^, we also tested the summer-temperature reconstructions for significance using the RandomTF test from the R package ‘palaeoSig’ and found that 528 of 1,744 reconstructions were substantial (*P* < 0.1). However, this significance test is rather conservative and there are several reasons for the creation of type II errors (false negatives), including a low diversity of taxa, a small number of sub-fossil observations, an input climate signal that is less variable or an inadequate training set^57^. Accordingly, a higher *P* value does not necessarily mean that the summer temperature has not been recorded, but rather that the information is insufficient to confirm it. The power of the RandomTF test can be low, as it is only sensitive to reconstructions with strong trends^31^. The use and caveats (particularly how it is prone to false negatives) of this test are debated and discussed in the literature^58–64^. Notably, in ref. ^65^, no correlation was found between the RandomTF *P* values and transfer function performance.

We also found no difference in performance based on the RandomTF test results. We reproduced the replicability analysis described above, but dividing the records into two groups of ‘significant locations’ (*P* < 0.1) and ‘not significant locations’ (*P* > 0.1). We would expect a clear improvement in odd–even replicability for the former if the test is skilful, but instead we find similar correlations (*r* = 0.52 and *r* = 0.54, respectively). Furthermore, the two groups are well-correlated together (*r* = 0.54), indicating they are recording the same millennial variability signal.

Therefore, instead of unduly discarding most records, we decided to include all records in the main analysis. We show that our conclusions are robust and continue to hold even if we restrict our analysis to the ‘significant locations’ only (*P* < 0.1), which yielded similar results to the ‘not significant locations’ (*P* > 0.1) (Supplementary Fig. 3).

### Testing for anthropogenic impacts

We considered all series entirely covering 0–8 ka and defined two temporal windows: the more recent (0–4 ka) and the more distant (4–8 ka) past. The 1,000–2,000-yr timescale band was taken to calculate the variance ratio between the two 4-kyr windows. We only included those series in our analyses whose spectral estimates covered at least one-fifth of an order of magnitude (on a base-10 logarithmic scale) for both time periods, a criterion that was met by 859 records. We found no systematic variance increase in the more recent half of the series. In fact, the more recent period, where human impacts may have contributed to an increased variability, is ~10% less variable than the earlier one. Similarly, the spatial distribution (Supplementary Fig. 2) did not show any obvious spatial patterns that could be related to human occupation, displaying a non-significant Moran’s *I* of 0.016 (*P* > 0.1; ‘Moran’s I’). If human occupation was the dominant driver of millennial-scale variability, we would have expected to observe an increase in variability over both Europe and China, where human occupation has been increasing the most over the last 4,000 years compared to the preceding 4,000 years. We thus conclude that human impacts on vegetation did not have a substantial enough impact on the slow variability to systematically bias millennial-scale variability estimates.

### Instrumental data

**Extended Data Fig. 10:**
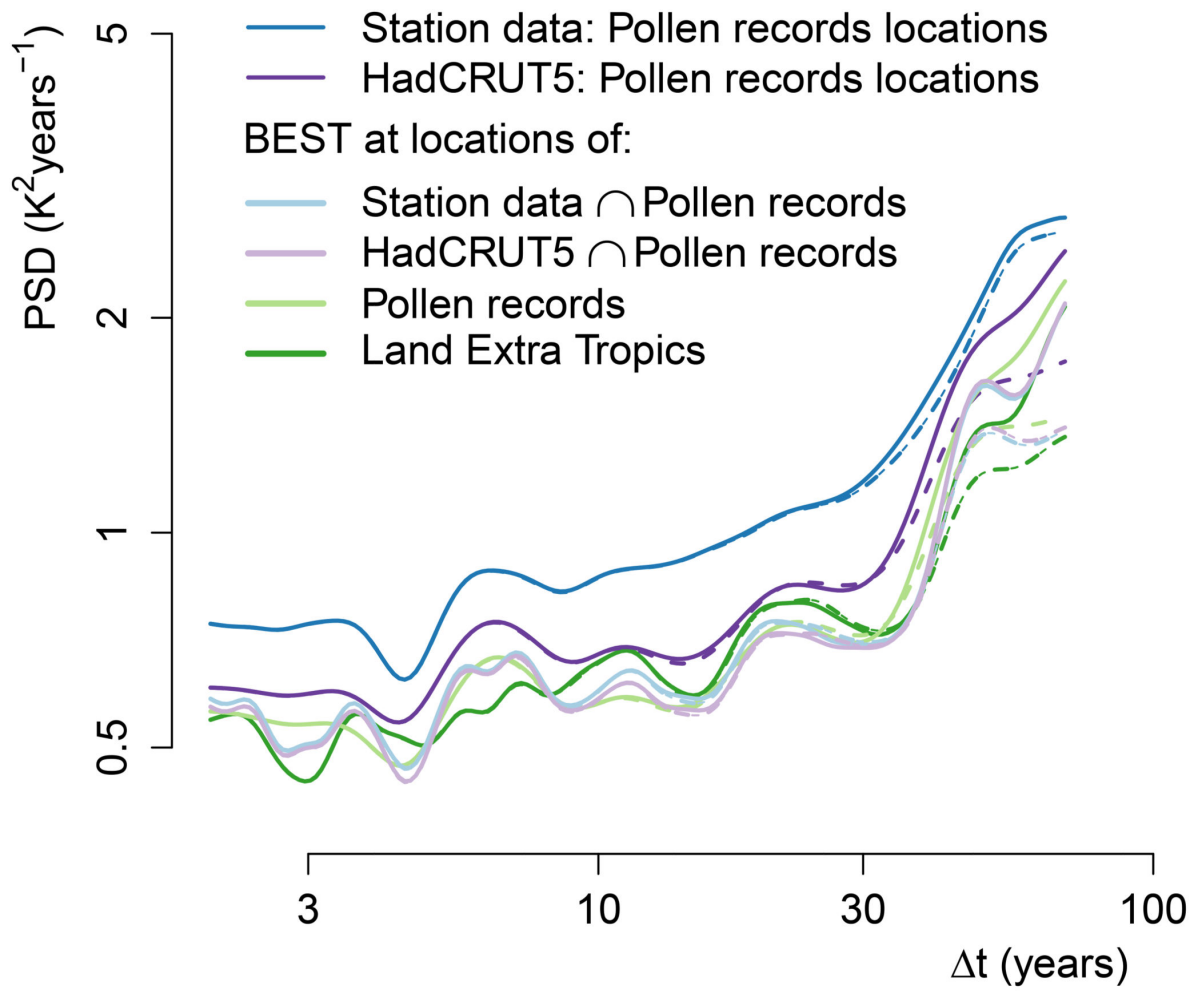
Local spectra of summer temperature for the noninfilled HadCRUT5 and the BEST interpolated products for varying spatial coverage. The average spectrum of the non-infilled HadCRUT5^66^ (dark purple) and the BEST^34^ station data (with at least 80 years of data, dark blue) overlapping with the pollen record locations (less than 300 km away in the case of the station data) are compared with the average spectrum of the BEST interpolated product for varying spatial coverage: at the pollen records locations (pale green), for all land extra tropic north of 20°N (dark green), and at the locations of overlap (legend uses the intersection symbol ⋂) between the pollen records with the noninfilled HadCRUT5 dataset (pale purple), and with the station data (light blue); logarithmically spaced axes were used. This supports that in all cases the spatial coverage is broad enough to be representative of the expected behaviour for the northern hemisphere land extra tropics and that HadCRUT5 is not affected by a coverage bias in Fig. 1 The higher variability in both station data and HadCRUT5 suggests that the infilled BEST product is biased low.

We considered two instrumental datasets—HadCRUT5^66^ and the Berkeley Earth Surface Temperature (BEST)^34^ land and ocean product—both covering the period 1850–2020. As an additional test, the breakpoint-adjusted monthly station data provided by BEST were considered. The BEST dataset has the advantage of providing full spatio-temporal coverage at a high spatial resolution covering all pollen locations. On the other hand, it appears that the spatial autocorrelation model assumed for the interpolation leads to a bias low in variability compared to the non-infilled HadCRUT5 and the station data (Extended Data Fig. 10). The choice of the optimal instrumental dataset is thus a trade-off between minimizing variance biases from the reconstruction method and minimizing the spatial sampling bias. Both datasets were used for the global analysis (Fig. 1) because the spatial sampling bias of HadCRUT5 is not important for the average spectrum of extra-tropical land regions (Extended Data Fig. 10). However, because it is important for spatial comparisons with the reconstructions (Figs. 2 and 3) to have both datasets at the same location, BEST was used as it provides spatial coverage of all the pollen record sites. The BEST equal area product was used for calculations, and the regular 1° × 1° product was used for visual display. The instrumental data were detrended from anthropogenic influences to a first-order component^67^ proportional to historical timeseries of doublings in atmospheric carbon dioxide concentration (that is, log(CO_2_))^68^. As this detrending might lead to a negative bias on the lowest frequencies, we show the series with and without log(CO_2_) detrending in Fig. 1, but generally perform the detrending for the subsequent results. We note that all our analyses are robust to these choices, or to the use of alternative instrumental datasets^69^.

As an additional test of the increase in variability at longer timescales that is shown in pollen and tree-ring records, we also considered long instrumental timeseries from the BEST station data. We identified 70 series that covered more than 170 years (the length of the gridded product) of summer-JJA temperature with gaps of at most five years. They further support the scaling increase at multi-decadal timescales, in agreement with the dendrochronological data (Extended Data Fig. 4).

### Model data

Three model simulations of the recent Holocene were considered: IPSL^35^, ECHAM5^36^ and CCSM3^70^. The first two are recent Holocene transient simulations of the past 6,000 years, and the latter is the TraCE-21ka deglaciation experiment^37^. We only retained the last 8,000 years of TraCE-21ka because it is comparable to the recent Holocene transient simulations, containing no more freshwater forcing events, which were the main drivers of deglaciation. We selected the average 2-m summer air temperature (JJA) and averaged it annually.

Because the long-term trends in summer temperature are linked to the precession in Earth’s orbit, we also analysed the timeseries after detrending for a 23-kyr sinusoid rather than using the standard linear detrending performed before computing spectral estimates. This approach attempts to minimize power leakage from the orbital forcing frequencies onto the observed frequencies (Fig. 1a, lines). The reduction in leaked power was not nearly as important in the case of the pollen-based reconstructions (Fig. 1a and Extended Data Fig. 5).

### Spectral estimates

The PSD estimates were calculated using the multitaper method^71^, adapted for irregular sampling through linear interpolation^72^, with number of tapers *n*_tapers_ = 3 and time-bandwidth parameter *ω* = 2, which yield up to *n*_tapers_ × *ω* = 6 degrees of freedom for the individual spectral estimates. Standard linear detrending was performed before computing the multitaper spectrum. Only timescales greater than twice the maximal resolution were kept, in order to minimize power loss due to the interpolation^47^. When averaging several spectra from timeseries with different temporal resolution (and thus different timescale/frequency sampling), the spectra were first linearly interpolated on a common timescale axis and the arithmetic mean taken. The PSD estimates were smoothed using a Gaussian kernel with a constant width of 0.03 in the logarithm (base 10) of the timescale^73^.

The confidence intervals were derived from the chi-squared distribution (*χ*^2^) of the PSD estimates. For the mean spectra, the degrees of freedom of each spectral estimate account for the frequency-dependent smoothing kernel and the number of individual spectra, limited by the expected effective spatial degrees of freedom at a given timescale, Given that the spatial coverage of our results is limited to extra-tropical land regions, we assumed a conservative value of a maximum of five effective spatial degrees of freedom for all time-scales^74^. The degrees of freedom of the *χ*^2^ distribution were estimated by moment-matching from an ensemble of surrogate data consisting of 1,000 realizations of fractional (or power-law) noise^75^, each time simulated with the estimated scaling exponent of the spectrum.

The mean spectra are more representative of the regions most sampled by the pollen records. To obtain comparable estimates, the instrumental and model timeseries were extracted at the same locations and processed in the same way. The mean local spectra of instrumental summer temperature at the location of pollen records and for the entire extra-tropical land regions (Extended Data Fig. 10, light and dark green) are very similar, thus supporting that the space sampled by the pollen records is representative for the extra-tropical land regions.

We conducted a sensitivity study to test the effect of potential non-climatic noise in reconstructions of the spectral estimates (Fig. 1). We assumed that the main noise sources, from aliasing and from the finite number of pollen grains and a misspecification of pollen taxa, are uncorrelated between samples (‘white’). To do this we computed the expected spectrum of a given record when replacing the temperature signal by a white noise and subtracted it from the reconstructions^9^. The confidence interval in Fig. 1 includes the *χ*^2^-based confidence interval of the spectrum of the original reconstructions and that of the reconstructions when subtracting a noise level of 0.5 K from all records. This shows that the uncertainty is highest on the faster timescales, as here the relative effect of the noise is largest. The provided confidence intervals should be a conservative bound, as the noise level of 0.5 K corresponds to more than twice the median spread of the leave-one-out cross-validation ensembles.

### Surrogate data and time uncertainty

To test the robustness of local spectral estimates to time uncertainty and irregular sampling in the pollen records, we produced surrogate data using the TraCE-21ka model simulation^37^. The simulated summer-JJA temperature was extracted at the location of pollen records and degraded to the resolution of the corresponding pollen records by sub-sampling the series after a low-pass filter with a characteristic timescale of twice the mean resolution^75^. In this way, a database analogous to the pollen database was created. We then introduced time uncertainty based on the statistical uncertainty from the radiocarbon dating of the Bacon age models^76^. The pollen database has a median time uncertainty of 250 yr, which is too optimistic^77^, as it does not take into account other effects such as, for example, uncertainty in the reservoir age, temporal variations in the reservoir age and the effect of pre-aged material and dated material not being representative of the sediment layer because of sediment mixing. We thus conducted a sensitivity test by amplifying the uncertainty around the mean age model of each record by factors of √2 and further added a random offset to each core to model the reservoir age effect, each time drawn from a normal distribution with a mean of 500 years and a standard deviation of √2 × 250 years. As the uncertainties add in quadrature, we obtained a database with a time uncertainty of 500 years. This is a realistic, but possibly optimistic, estimate according to the few studies comparing radiocarbon-based lake chronologies to independent constraints from widespread ecological events or to independent dating techniques^78–80^.

In analogy to the spectral estimation of the true pollen timeseries, we estimated the average local spectra of temperature variability from the time-uncertain surrogate timeseries. The resulting mean spectra are consistent between the original annual model data and the surrogate databases with and without time uncertainty (Extended Data Fig. 1), showing that our method is robust to irregular resolution of our timeseries and realistic estimates of time uncertainty.

To study the spectrum of spatial means, we computed the average of the surrogate timeseries with and without time uncertainty after interpolating each series to a 100-yr resolution following ref. ^5^. The spectra of average (hemispheric) temperature show strong power loss at all timescales due to the irregular resolution and time uncertainty (Extended Data Fig. 1). Only the variability at the longest timescales is preserved, as the long-term trend is robust to these effects. This shows that the amplitude of variability from mean timeseries over large spatial scales cannot be quantitatively interpreted without knowledge of the amount and structure of time uncertainty.

### Variance ratios

Variance ratios were computed by taking the ratio between the mean PSD over the same timescale band between different series after interpolating in the spectral domain. Because the ratio of two *χ*^2^-distributed variables follows an F-distribution, the ratios were multiplied by (*d* – 2)*d*^−1^, where *d* is the number of degrees of freedom of the denominator^27^.

### Sub-decadal variability binning

The data were aggregated based on the mean sub-decadal variability PSD_2–10 years_, defined as the mean PSD over the 2–10-yr band. We calculated PSD_2–10 years_ for each of the 624 instrumental grid points for which pollen records were present nearby, ordered the results, and split them into eight non-overlapping bins (Extended Data Fig. 7c,d). Each pollen record was assigned to the nearest instrumental grid point and averaged in the spectral domain. Varying the number of bins, for example, using 20 bins instead of 8, led to similar correlations. The standard errors of the millennial variability estimates were used as weights for the correlation calculation (using Pearson’s correlation) and for the visual representation in Fig. 3b,c. The *P* values were calculated taking into account the spatial autocorrelation of the fields and the binning process. We performed Monte-Carlo experiments using surrogate fields generated based on the randomization of the phase of the two-dimensional Fourier transform^81^. Ten thousand surrogate fields were thus generated for PSD_2–10 years_ and *β*_10–60 years_, and the analysis was repeated to obtain the correlation coefficients after binning, from which the *P* values were calculated based on the empirical quantile. The significance is not sensitive to the number of bins (Supplementary Fig. 4).

### Relative land influence index

As a further test of the relationship between millennial variability and marine influence, PSD_1,000–3,000 years_ was compared to the RLI metric developed by McKinnon and colleagues^46^ (Extended Data Fig. 9). The RLI quantifies the relative contributions of land and ocean to atmospheric fluxes at a given location using a Lagrangian trajectory model and reanalysis data. The expected relationship, less millennial variability for higher RLI, is observed, except for the very high RLI values (RLI > 0.78, indicated in red in Extended Data Fig. 9a). This is further evidenced by binning the reconstructions according to RLI into eight bins of 198 records for those with RLI < 0.78, and a further ninth bin with the 158 records with RLI > 0.78 (Extended Data Fig. 9b). The latter contains records in Siberia, Central Asia and East Asia where the RLI is highest (Extended Data Fig. 9c), but the millennial variability remains high (except for the records in Siberia, which follow the expected trend). This could either be related to strong local climate sensitivity, which may be linked to hydrological feedback due to the arid conditions^42^ or to the snow-albedo feedback at higher elevations^45^, and to a bias in the pollen-based temperature estimates from precipitation-sensitive vegetation in this region^29^. Also, the discrepancy between RLI and the sub-decadal variability over East Asia suggests that RLI might not be an appropriate metric of continentality there. Notably, East Asia is one of the few regions where an EBM using RLI as a mixing parameter does not reproduce the seasonal gain (associated to continentality) skil-fully^25^. The *P* value was obtained in the same way as for the sub-decadal variability binning, accounting for the spatial autocorrelation and the distribution of the fields.

### Moran’s *I*

Moran’s *I* spatial autocorrelation index was calculated using the method from ref. ^82^ as implemented in the R-package ‘ape’^83^. The weight matrix used corresponds to the inverse of the distance between sites.

## Extended Data

## Supplementary Material

Source Data File Figure 1

Source Data File Figure 2

Source Data File Figure 3

Supplementary Information

## Data Availability

The pollen-based reconstructions data that support the findings of this study are available in PANGEA with the identifier https://doi.pan-gaea.de/10.1594/PANGAEA.930512. The BEST instrumental data are available from www.berkeleyearth.org/data. The HadCRUT instrumental data are available from https://crudata.uea.ac.uk/cru/data/temperature/#datdow. The marine proxy data are available in PANGEA with the identifier https://doi.org/10.1594/PANGAEA.899489. The IPSL transient simulation was run as part of the JPI-Belmont project PACMEDY (ANR-15-JCLI-0003-01) and is available upon request from pascale.braconnot@lsce.ipsl.fr. The ECHAM5 transient simulation data are available in PANGEA with the identifier https://doi.org/10.1594/PANGAEA.773607. The TraCE-21ka simulation data are available from the Climate Data Gateway at NCAR https://www.earthsystemgrid.org/project/trace.html. The CMIP5 millennium simulations are available through the Earth System Grid Federation portal https://esgf-data.dkrz.de. The dendrochronological data can be accessed as part of the PAGES2k database at http://wiki.linked.earth/PAGES2k#Downloads. Source data are provided with this paper.

## Code availability

The R code is publicly available in Zenodo with the identifier https://doi.org/10.5281/zenodo.7062762. The R package RScaling^75^ is available in Zenodo with the identifier https://doi.org/10.5281/zenodo.7062678.
