# Supplementary material for: Millennial-scale climate variability over land overprinted by ocean temperature fluctuations
Source: Nat Geosci. Author manuscript; Available in PMC 2023 Apr 30. (PMC7614181; doi:10.1038/s41561-022-01056-4)
Supplement: Supplementary Information [file EMS154709-supplement-Supplementary_Information.pdf]

nature geoscience

Article

https://doi.org/10.1038/s41561-022-01056-4

Millennial-scale climate variability over land overprinted by ocean temperature fluctuations

In the format provided by the
authors and unedited

Supplementary Information

Note 1: Tree Ring Data Analysis

The dendrochronological series were extracted from the PAGES2k database[1]. The tree ring width (TRW) timeseries are the most abundant type, with 353 timeseries from the northern hemisphere. In addition, there are 58 maximum latewood density (MXD) timeseries, mostly located in North America. The spectral estimates were computed on the original TRW and MXD timeseries before calibrating them for temperature (in kelvins) using the closest instrumental record. The calibration was carried out in the spectral domain by making the power spectral density of the dendrochronological spectra equal to the closest instrumental spectral estimates across their shared timescales. The calibration in the spectral domain corresponds to the standard variance matching step in Pairwise Comparison (PaiCo) or Composite plus Scale (CPS)[2], except that instead of matching the variance over the target instrumental period which is only 30 years long, we match the variance over the whole timeseries (in the overlapping frequency range) resulting in a more robust variability matching (but assuming the statistics are stationary).

Temperature calibrated dendrochronological MXD and TRW timeseries provide strong support for our interpretation of climate variability over land derived from the instrumental record and the pollen-based reconstructions, albeit with more restricted spatial coverage. The resulting average spectra for the MXD and TRW timeseries both scale with $\beta \approx 0.9$ over 20-200 years. For $\Delta t \geq 200$ years, the MXD-based spectrum follows the pollen-based result, while the TRW-based spectrum loses power, a bias expected from the growth-trend removal[3]. On the other end, for $\Delta t \leq 20$ years, the shape of the MXD-based spectrum flattens in agreement with the instrumental data, while the TRW-based spectrum starts to exhibit a red bias, as previously recognized by Franke et al. (2013)[4], which probably stems from the temporal autocorrelation induced by the biological response of the tree growth to changing climatic conditions[5]. In agreement with previous studies, our analysis also supports that MXD is indeed a more reliable temperature proxy than TRW[6].

Note 2: Energy-Balance Equations

We consider a simple energy-balance model such as MAGICC6 which has been shown to have the capacity to emulate complex GCMs for global average temperature[7]. In this formulation, the Earth's atmosphere is divided into 4 compartments, two over land and two over the ocean (one per hemisphere). The ocean itself is then divided into a number of layers, distinct for each hemisphere, which can store energy. The over-land atmospheric box in the Northern Hemisphere is connected to the over-ocean atmospheric box in the same hemisphere, which is in turn connected to the mixed-layer (oceanic surface layer) in the Northern Hemisphere and the over-ocean atmospheric box in the Southern Hemisphere.

Following Geoffroy et al. (2015)[8] and Frederiksen and Rypdal (2017)[9], we assume that the temperature over the ocean in the Northern Hemisphere ΔT_O is only proportional to the mixed-layer temperature in the Northern Hemisphere ΔT_{SST}, i.e. $\Delta T_O = a \Delta T_{SST}$, thereby neglecting any cross-equatorial atmospheric heat transport. The proportionality constant a accounts for the greater

warming in the atmosphere with respect to the mixed-layer; it is also greatly influenced by the amount of sea-ice cover[10]. The temperature evolution over land in the northern hemisphere ΔT_L can thus be written as :

$$C_L \frac{d\Delta T_L(t)}{dt} = -\lambda_L \Delta T_L(t) + F_L(t) + k(\mu a \Delta T_{SST}(t) - \Delta T_L(t))$$

where C_L is the heat capacity of the atmosphere over land, λ_L is the land feedback parameter, $F_L(t)$ is the forcing over land, k is a coefficient for the heat exchange between land and ocean which also accounts for atmospheric (radiative) feedbacks, and μ is the asymmetric heat exchange coefficient between land and ocean. Given that the heat capacity over land is small, we can write an approximate expression assuming C_L=0:

$$\Delta T_L(t) = S_L F_L(t) + \Omega S_L \Delta T_{SST}(t)$$

where we defined a new land sensitivity coefficient $S_L^{-1} = k + \lambda_L$ and a land-ocean coupling coefficient $\Omega = k a \mu$. If the coupling coefficient k is weak ($k \to 0$), then the oceanic term vanishes altogether, whereas if the coupling is strong ($k \to \infty$), the forcing term vanishes and only the oceanic term matters. The forcing over land $F_L(t)$ is generally taken as a random white noise forcing due to the relatively fast atmospheric processes[11]. Assuming zero heat capacity over land, the white noise forcing directly translates into a flat spectrum (β=0) for the inter-annual macroweather variability. In practice, the small over-land heat capacity introduces a memory which explains the weak inter-annual scaling of the local temperature spectra (β≈0-0.3)[12]. The average land temperature in the Northern Hemisphere can therefore be described as a linear combination of an instantaneous response to random forcing and a component proportional to the SST.

By taking the squared Fourier transform of both sides we obtain an expression for the power spectrum :

$$\left|\Delta \tilde{T}_L(\omega)\right|^2 = S_L^2 \left|\tilde{F}_L(\omega)\right|^2 + \Omega^2 S_L^2 \left|\Delta \tilde{T}_{SST}(\omega)\right|^2 + S_L^2 \Omega \left|\tilde{F}_L(\omega) \Delta \tilde{T}_{SST}(\omega)\right|$$

If the forcing over land is independent from the SST, then the mixed term vanishes and we are left with the power spectrum of the temperature over land as a linear combination of the power spectra of the forcing term and the SST:

$$\left|\Delta \tilde{T}_L(\omega)\right|^2 \approx S_L^2 \left|\tilde{F}_L(\omega)\right|^2 + \Omega^2 S_L^2 \left|\Delta \tilde{T}_{SST}(\omega)\right|^2$$

The two terms can be assumed to be approximately independent since the one proportional to SST is the result of heat diffused from the ocean to the land, whereas the time-dependent forcing over land term is mostly the result of local radiative imbalance and weather instabilities, which are not expected to be correlated to the large-scale heat transport.

Note 3: Estimation Uncertainty of the Multi-Decadal Scaling Exponent

In the average spectrum shown in Fig. 1 of the main text, the change of scaling behaviour from the rather flat sub-decadal timescales appears mild, yielding an estimated multi-decadal scaling exponent $\beta_{10\text{-}60\text{ years}} \approx 0.6$. In contrast, if we assume the piecewise power-law scaling model (shown on Fig. 1b of the main text) fitted to both instrumental data (over 2-10 years timescales) and pollen-based reconstructions (over 1000-3000 years timescales), then we obtain $\beta_{10\text{-}60\text{ years}} \approx 0.9$.

The difference in scaling might be random, i.e. our instrumental record just happened to sample a lower variability 170-year period. To show this, we simulate a 170-ka long annual timeseries with the temporal covariance structure following the piecewise power-law scaling model as our null hypothesis. We then compute the power spectrum of 1000 non-overlapping 170-year pieces of this timeseries. We randomly select 5 members and average their spectra together before estimating the multi-decadal scaling exponent, and this way we obtain the distribution of $\beta_{10\text{-}60\ years}$ values assuming 5 effective spatial degrees of freedom (Fig. S1a,b). The distribution shows the large uncertainty of estimating $\beta_{10\text{-}60\ years}$ (90% confidence interval of [0.5,1.2]) from the relatively short instrumental record as well as the effect of the log CO_2 detrending and the low bias of the multitaper method on the lowest frequency[13]. The scaling exponent estimated from the instrumental data $\beta_{10\text{-}60\ years} \approx 0.6$ is within the distribution of scaling exponents from the ensemble of 170-year long pieces and we thus can't reject the null hypothesis that the instrumental data reflects our piecewise power-law scaling model.

For comparison, we perform the same experiment on the model simulations, splitting the 6000 year (8000 years in the case of TraCE-21ka) in 170-year segments allowing for 60 years of overlap to increase the number of realizations. We then obtain 90% confidence intervals for the multi-decadal scaling exponent $\beta_{10\text{-}60\ years}$ of [-0.12,0.47], [-0.15,0.31] and [-0.15,0.29] for ECHAM5, IPSL and TraCE-21ka respectively, thus showing a similar estimation uncertainty as our surrogate experiment and supporting that the scaling of the instrumental data is higher than in the model simulations.

There is also uncertainty in the specification of the piecewise power-law scaling model since the variability in the BEST instrumental data used is likely biased low (Extended Data Figure 10), and the reconstructions include a certain amount of non-climatic noise leading to increased variability. Fitting the model to the non-infilled HadCRUTv5 and the pollen-based reconstruction with 0.5K of noise subtracted, we obtain a better agreement with an average $\beta_{10\text{-}60\ years} \approx 0.7$. (90% confidence interval of [0.3,1.0]; Fig. S1c,d). As the noise correction is only a sensitivity experiment rather than a best estimate (see Spectral Estimate Method Section), we prefer to show as the main result the (imperfect but without additional assumptions) non-corrected version. Ultimately, the model fitting is heuristic and we leave for future work a more rigorous and spatially-detailed estimation.

Supplementary Figures

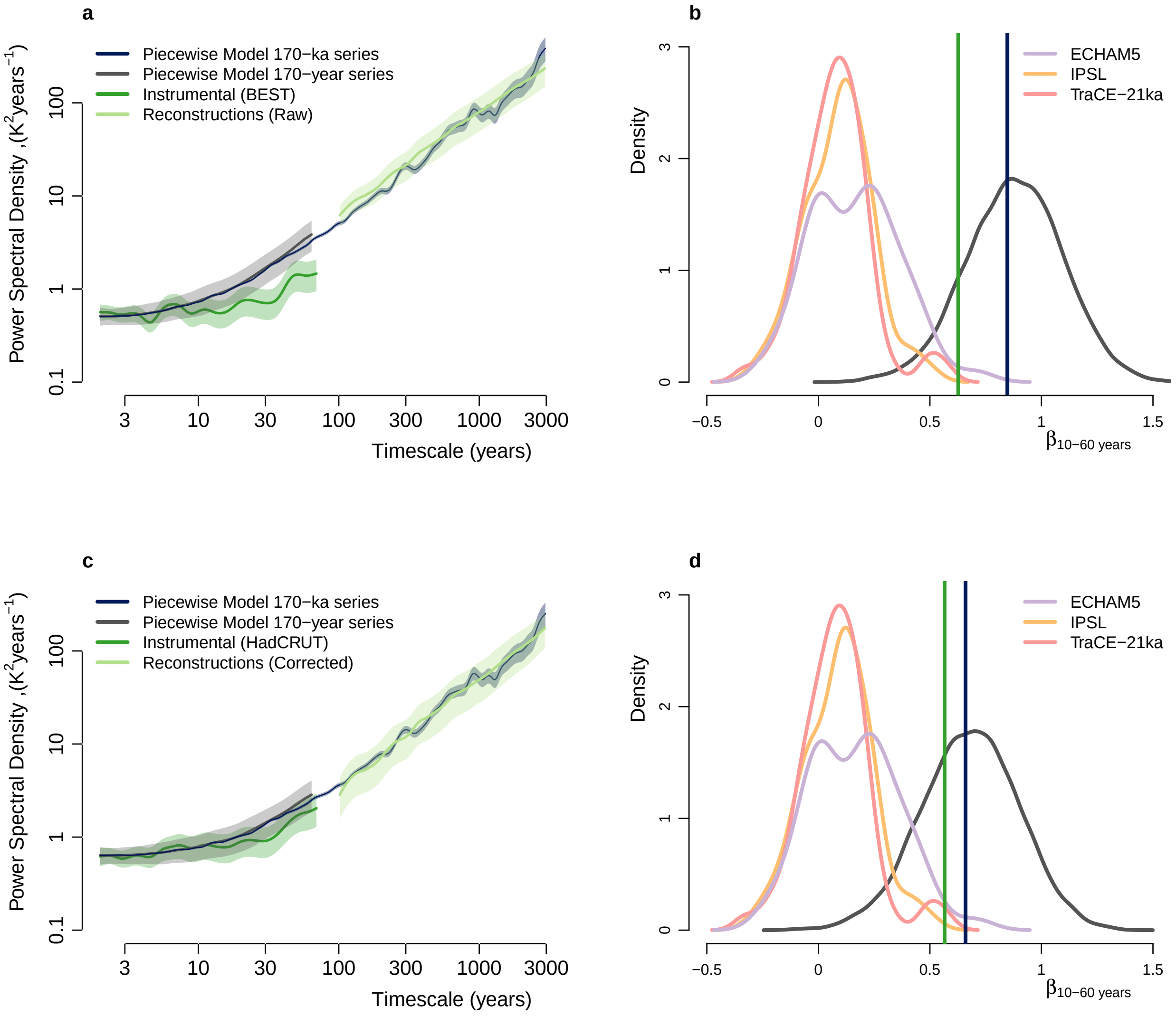

Figure S1 | Estimation uncertainty of the multi-decadal scaling exponent. a. Comparison of power spectra from BEST instrumental data, pollen-based reconstructions and surrogate timeseries based on the piecewise power-law scaling model. For the latter, the estimate from a long 170-ka realization and the mean estimate and 90% confidence interval from the ensemble of 1000 170-year long sub-segments are shown. **b**. The distribution of the multi-decadal scaling exponent calculated from the 1000 non-overlapping segments is shown along the estimates from the full 170-ka series and the BEST instrumental data (vertical lines with colours corresponding to legend on **a**). Also shown are the analogous distributions calculated from the climate model simulations. **c**. Same as **a**, but using the HadCRUT instrumental data and the reconstructions with a 0.5 K noise level removed (see Spectral Estimates Method Section). **d**. Same as **b**, but using the data shown on **c**.

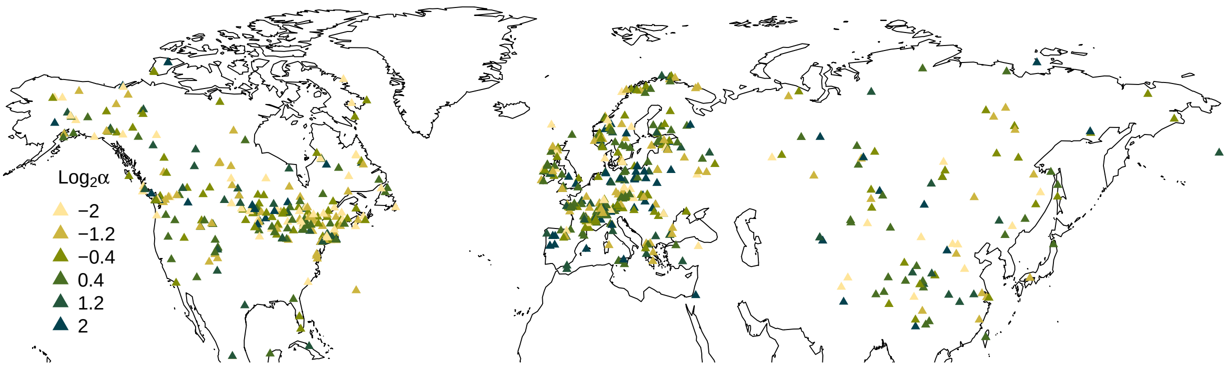

Figure S2 | Variance ratio between late and mid-Holocene temperature variability. Shown are the $\log_2$ of the variance ratios α (i.e. the number of doublings) for the 1000-2000 years timescale band between the late Holocene (4ka-0ka BP) and the mid-Holocene (8ka-4ka). A positive $\log_2 \alpha$ implies that the later period, where human impacts should be more important, was more variable.

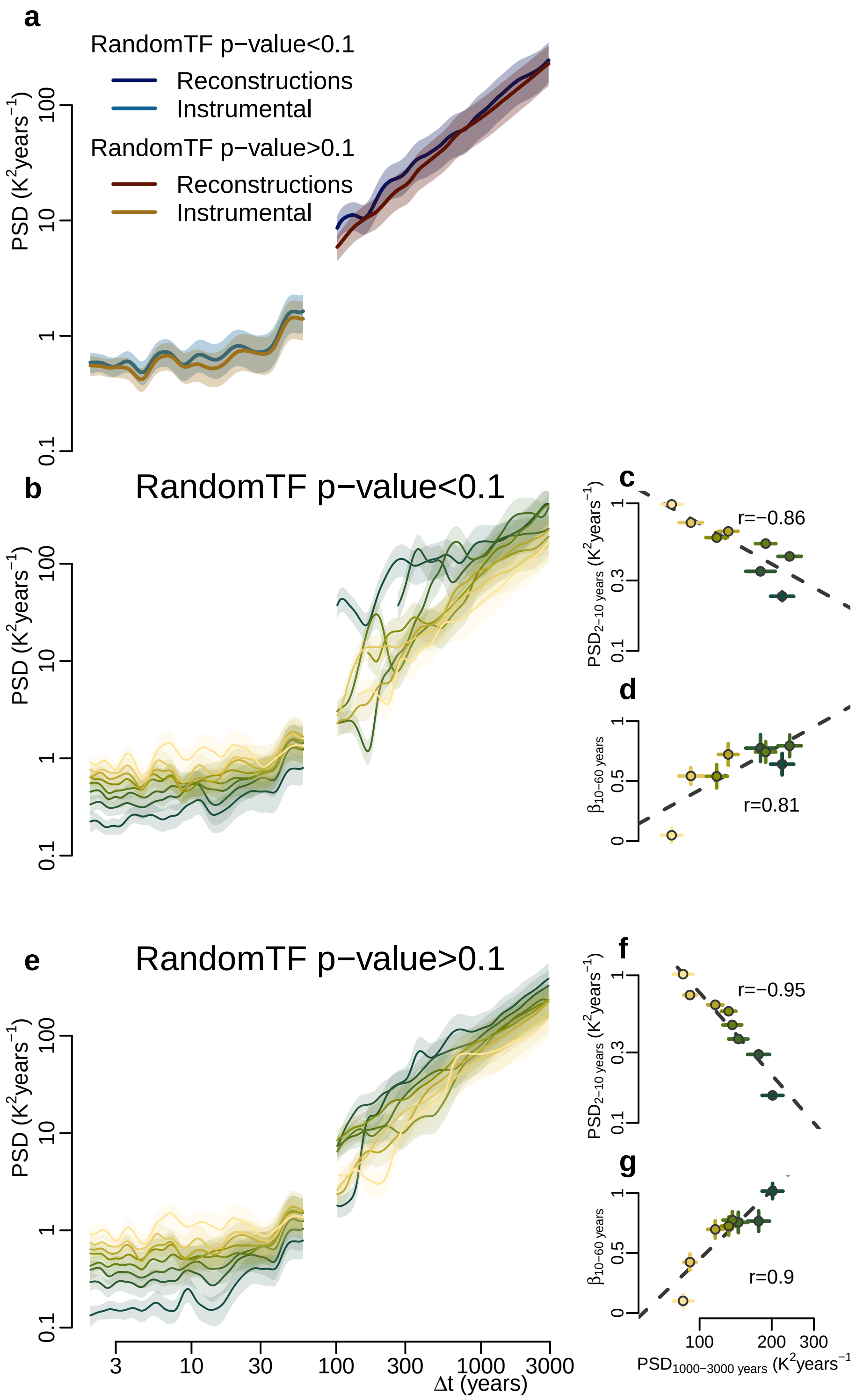

Supplementary Figure S3 | Comparison of pollen-based reconstructions based on the outcome of the RandomTF test. A total of 1744 pollen records that provided spectral estimates were tested for statistical significance using the RandomTF test (see Methods) and separated into 'significant locations' (P<0.1, one-sided, n=528) and 'not significant locations' (P>0.1, one-sided, n=1216). **a,** Same as Fig. 1a, but for the significant and not significant locations separately. For simplicity, the model simulations spectra are not reproduced and only the BEST instrumental spectra for the corresponding locations are shown. Shading indicates 90% confidence intervals around the mean. **b,c,d** Same as Fig.3, but for the 'significant locations' only. **e,f,g** Same as Fig.3, but for the 'not significant locations' only. Logarithmically spaced axis were used to display the spectra and the PSD.

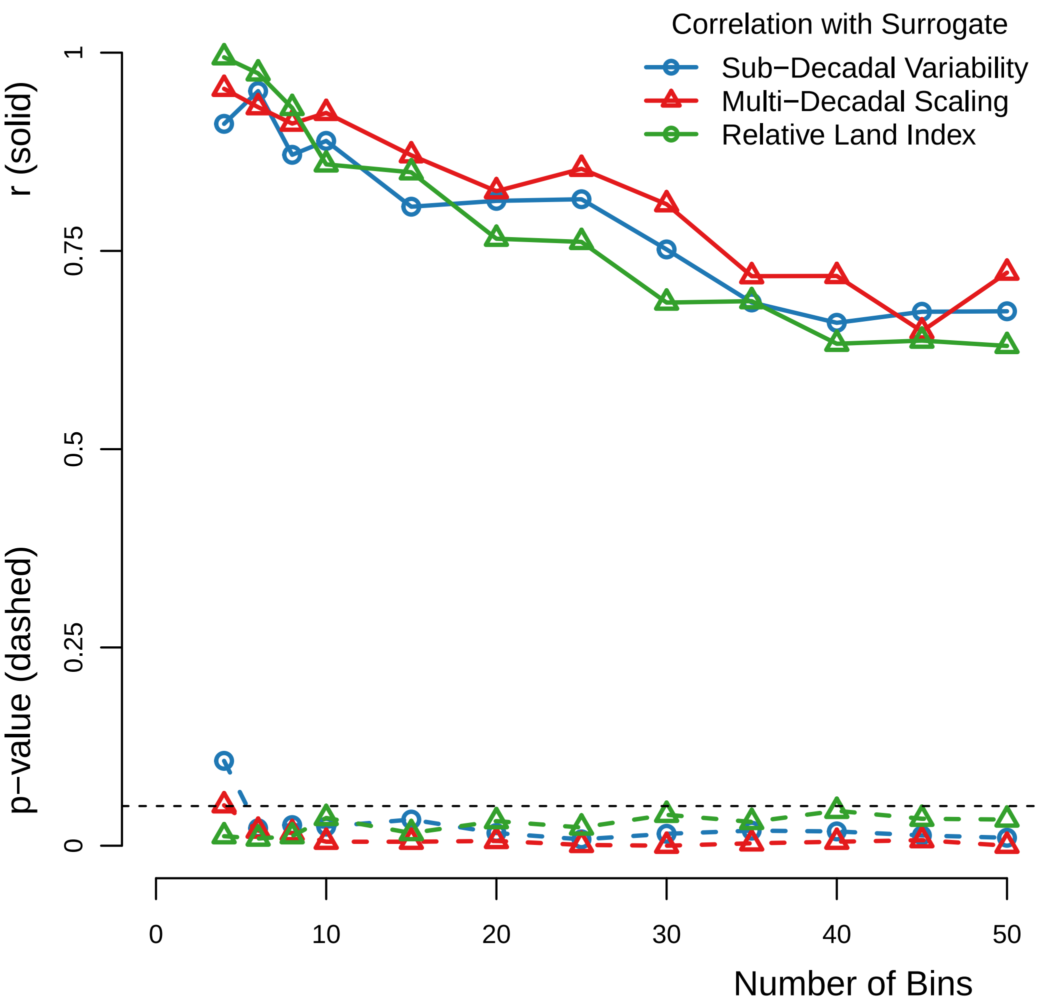

Supplementary Figure S4 | Sensitivity of the correlation and significance between sub-decadal variability, multi-decadal scaling and relative land influence index with millennial variability on the number of bins. We perform the analysis shown on Fig. 3, but for varying number of bins. We extract the correlation r (solid) and the associated p-value (dashed) for each bin size based on a one-sided test using empirical quantiles derived from a Monte-Carlo experiment using surrogate fields generated based on the randomization of the phase of the 2D Fourier transform (n=10000; see Methods Sub-Decadal Variability Binning). The P = 0.05 significance level is indicated (horizontal dashed line).

References

1. PAGES2k Consortium *et al.* A global multiproxy database for temperature reconstructions of the Common Era. *Sci. Data* **4**, 170088 (2017).
2. Ahmed, M. *et al.* Continental-scale temperature variability during the past two millennia. *Nature Geosci* **6**, 339–346 (2013).
3. Collins, M., Osborn, T. J., Tett, S. F. B., Briffa, K. R. & Schweingruber, F. H. A comparison of the variability of a climate model with paleotemperature estimates from a network of tree-ring densities. *J. Clim.* **15**, 1497–1515 (2002).
4. Franke, J., Frank, D., Raible, C. C., Esper, J. & Brönnimann, S. Spectral biases in tree-ring climate proxies. *Nat. Clim. Change* **3**, 360–364 (2013).
5. Frank, D., Büntgen, U., Böhm, R., Maugeri, M. & Esper, J. Warmer early instrumental measurements versus colder reconstructed temperatures: shooting at a moving target. *Quat. Sci. Rev.* **26**, 3298–3310 (2007).
6. Wilson, R. *et al.* Last millennium northern hemisphere summer temperatures from tree rings: Part I: The long term context. *Quat. Sci. Rev.* **134**, 1–18 (2016).
7. Meinshausen, M., Raper, S. C. B. & Wigley, T. M. L. Emulating coupled atmosphere-ocean and carbon cycle models with a simpler model, MAGICC6 – Part 1: Model description and calibration. *Atmospheric Chem. Phys.* **11**, 1417–1456 (2011).
8. Geoffroy, O. *et al.* Transient Climate Response in a Two-Layer Energy-Balance Model. Part I: Analytical Solution and Parameter Calibration Using CMIP5 AOGCM Experiments. *J. Clim.* **26**, 1841–1857 (2013).
9. Fredriksen, H.-B. & Rypdal, M. Long-Range Persistence in Global Surface Temperatures Explained by Linear Multibox Energy Balance Models. *J. Clim.* **30**, 7157–7168 (2017).
10. Raper, S. C. B., Gregory, J. M. & Osborn, T. J. Use of an upwelling-diffusion energy balance climate model to simulate and diagnose A/OGCM results. *Clim. Dyn.* **17**, 601–613 (2001).
11. Hasselmann, K. Stochastic climate models Part I. Theory. *Tellus* **28**, 473–485 (1976).
12. Lovejoy, S., del Rio Amador, L. & Hébert, R. The ScaLIng Macroweather Model (SLIMM): using scaling to forecast global-scale macroweather from months to decades. *Earth Syst. Dyn.* **6**, 637–658 (2015).
13. Prieto, G., Parker, R., Thomson, D., Vernon, F. & Graham, R. Reducing the bias of multitaper spectrum estimates. *Geophys. J. Int.* **171**, 1269–1281 (2007).
